# Melatonin in Musculoskeletal and Spine-Related Conditions: A Systematic Review of Preclinical and Clinical Evidence

**DOI:** 10.3390/medicina62101946

**Published:** 2026-10-09

**Authors:** Michele Bisaccia, Barbara Bifarini, Umberto Ripani, Lorenzo Lucchetta, Edoardo Bonanno, Marco Siragusano, Dario Di Mitri, Francesco Bronzini, Giuseppe Rinonapoli

**Affiliations:** 1 Orthopedics and Traumatology Department, Medical School, University of Perugia, S. Maria della Misericordia Hospital, 06123 Perugia, Italy; lorenzo.lucchetta@outlook.it (L.L.);; 2 Anaesthesia and Resuscitation, Department of Medicine and Surgery, S. Maria Misericordia Hospital, S. Andrea delle Fratte, 06132 Perugia, Italy; b.bifarini@libero.it; 3 Division of Clinic Anaesthesia, Department of Emergency Hospital Riuniti, Conca Street 71, 60126 Ancona, Italy; umberto.ripani@ospedaliriuniti.marche.it

**Keywords:** melatonin, systematic review, osteoarthritis, osteoporosis, fracture healing, sarcopenia, tendon, spinal cord injury, intervertebral disk, sports recovery

## Abstract

*Background and Objectives:* Experimental studies suggest that melatonin can influence antioxidant, inflammatory, mitochondrial, and chronobiological pathways relevant to musculoskeletal degeneration, repair, and selected spine-related conditions, but clinical translation remains uncertain. This systematic review evaluated primary in vitro, animal, and human evidence concerning exogenous parent melatonin in these conditions. *Materials and Methods:* PubMed/MEDLINE and Europe PMC were searched from inception to 6 August 2026; ClinicalTrials.gov was mapped separately. Eligible reports were peer-reviewed comparative primary investigations. One reviewer performed screening, extraction, and a screen-level methodological limitation assessment, followed by a complete consistency pass; no automation tools were used. Complete articles were consulted where accessible, but access status was not logged prospectively and the exact number accessed cannot be reconstructed reliably. Because quantitative pooling was not appropriate, evidence was synthesized by stream and clinical domain according to PRISMA 2020 and SWiM. The review was not registered, and no standalone protocol was prepared. *Results:* The searches yielded 3201 records and 1646 unique records after deduplication. Of 437 reports taken forward for retrieval and report-level assessment using available information, 208 were included (23 in vitro, 142 animal, and 43 human reports). Preclinical studies generally reported osteogenic, chondroprotective, antioxidant, anti-inflammatory, mitochondrial, and tissue-repair effects, but findings were not uniformly favorable: high-dose fracture-remodeling impairment and MT2-dependent heterotopic ossification were reported. Human evidence was limited, heterogeneous, and showed frequent screen-level methodological limitations. Formulations, routes, and exposures were non-equivalent. *Conclusions:* Melatonin is biologically plausible but clinically unproven in the reviewed conditions. No formulation, dose, or route can presently be recommended for disease modification, neurological recovery, or tissue regeneration.

## 1. Introduction

Melatonin, or N-acetyl-5-methoxytryptamine, is an evolutionarily conserved indoleamine best known for circadian regulation and synchronization of biological rhythms. The experimental and physiological literature has also investigated its relationships with redox balance, mitochondrial stability, inflammatory signaling, and immune responses [1,2,3,4,5,6,7]. These proposed actions are of interest in tissues exposed to chronic mechanical loading, relative hypoxia, senescence, metabolic stress, and repeated inflammatory stimulation, including bone, articular cartilage, skeletal muscle, tendon, ligament, and the intervertebral disk; they should not be interpreted as proof of therapeutic benefit.

Although pineal secretion remains the most recognized source of circulating melatonin, extrapineal synthesis has been reported in the gastrointestinal tract, retina, immune cells, and bone marrow. This distribution has generated the hypothesis that melatonin may also act through paracrine, autocrine, or intracellular signaling [6,7,8,9,10,11,12,13,14,15,16]. In musculoskeletal models, these signals have been studied in relation to mitochondrial dysfunction, oxidative damage, NF-κB activation, altered autophagy, impaired matrix turnover, and cellular senescence [17,18,19,20,21]. The clinical importance of these associations remains uncertain.

The burden of musculoskeletal disorders continues to increase with population aging. Osteoarthritis, osteoporosis, sarcopenia, tendinopathy, and degenerative spine disease share several biological denominators: persistent low-grade inflammation, excessive reactive oxygen species production, impaired tissue repair, reduced anabolic signaling, and disruption of the circadian and endocrine environment. The age-related decline in nocturnal melatonin secretion has therefore been proposed as one of several factors potentially associated with the increased vulnerability of musculoskeletal tissues in older adults, although a direct causal relationship has not been definitively established [17,18,19,20].

From a clinical perspective, established disease-specific treatment and rehabilitation remain central. Experimental cell and animal studies suggest that melatonin may influence oxidative stress, inflammation, mitochondrial function, cell survival, matrix metabolism, and circadian homeostasis [21,22,23,24,25,26,27,28,29,30,31,32,33,34,35]. These multi-pathway observations are mechanistic hypotheses, not evidence that melatonin is a panacea or a clinically effective treatment across conditions; most have not been confirmed in adequately powered human trials.

The spine-related human literature also requires cautious interpretation. Herdea et al. reported preliminary one-year findings in 51 children with adolescent idiopathic scoliosis who received a combined regimen of melatonin 1.5 mg/day, calcium 600 mg/day, and vitamin D 2000 IU/day [36]. Allocation by odd/even sequence, lack of investigator blinding, and the multicomponent intervention prevent attribution of the reported Cobb-angle difference to melatonin alone. The study is therefore discussed as contextual evidence and was not counted as proof of independent melatonin efficacy.

The objective of this systematic review was to identify, critically interpret, and synthesize primary evidence on the effects and safety of exogenous melatonin in musculoskeletal and spine-related conditions across in vitro, animal, and human studies. We additionally sought to determine the consistency of findings across evidence streams, identify areas in which preclinical findings have progressed to clinical evaluation, and define the principal limitations that prevent translation into routine practice. The review question was: in musculoskeletal or spine-related cells and tissues, experimental animals, and human populations, what biological, structural, functional, clinical, and safety effects are associated with melatonin compared with vehicle, no melatonin, placebo, usual care, or an active comparator?

## 2. Materials and Methods

### 2.1. Review Design and Reporting

This systematic review was prepared in accordance with the Preferred Reporting Items for Systematic Reviews and Meta-Analyses (PRISMA) 2020 statement [37]. Because predefined criteria for meaningful quantitative pooling were not met, the structured non-meta-analytic synthesis followed the Synthesis Without Meta-analysis (SWiM) reporting guideline [38]. The review was not registered, and no standalone protocol document was prepared. The decision to convert the originally submitted narrative review into a systematic review was made during peer review; before the updated search was run, the authors specified the PICOS criteria, evidence streams, data fields, screen-level methodological limitation domains, and qualitative synthesis plan. The post hoc conversion and subsequent methodological amendments are documented in Supplementary File S1, Section S5, and are acknowledged as limitations. Later editorial revisions did not alter the recorded search counts or inclusion decisions.

### 2.2. PICOS Framework

The population, intervention, comparator, outcomes, and study design (PICOS) framework guided eligibility assessment (Table 1).

### 2.3. Eligibility Criteria

Only primary studies published as full articles in peer-reviewed journals were eligible for the evidence synthesis. Studies were required to evaluate exogenous parent melatonin (N-acetyl-5-methoxytryptamine), administered systemically, applied locally, or incorporated into a delivery platform, in a prespecified musculoskeletal or spine-related model or population; include an eligible comparator; and report at least one relevant biological, structural, functional, clinical, or safety outcome. Spinal cord injury was retained as a separate neurological spine-related domain because immobilization-related bone loss, muscle atrophy, pain, and rehabilitation needs overlap with orthopedic practice; it was not classified as an osteoarticular disease, and its findings were not pooled with bone or joint evidence. Immediate-release, prolonged-release, synthetic, plant-derived, and carrier-loaded preparations were classified as formulations of parent melatonin, not as separate chemical analogs. Studies of other melatonergic agonists or analogues (including ramelteon, tasimelteon, and agomelatine), endogenous melatonin concentrations without an intervention, and multicomponent interventions from which the independent melatonin effect could not be separated were excluded from efficacy conclusions. Receptor antagonists such as luzindole or 4-P-PDOT were retained only when used as mechanistic probes within an otherwise eligible melatonin experiment. Conference abstracts, protocols, preprints, dissertations, book chapters, editorials, letters without original data, narrative reviews, systematic reviews, and meta-analyses were excluded from the primary synthesis. Registered trials without a peer-reviewed report were described separately and were not treated as evidence of efficacy.

### 2.4. Information Sources and Search Strategy

PubMed/MEDLINE and Europe PMC were searched from database inception to 6 August 2026. Controlled vocabulary and title/abstract terms for melatonin were combined with terms covering bone metabolism and repair, osteoporosis, fracture healing, cartilage and osteoarthritis, skeletal muscle, sarcopenia, tendon and ligament disorders, spinal cord injury, intervertebral-disk degeneration, orthopedic procedures, and exercise-related tissue stress. The exact executable strategies, platforms, search dates, limits, and database yields are reproduced in Supplementary File S1, Section S1. No language restriction was applied at the search stage. Reference lists and other websites were not used as independent identification sources.

ClinicalTrials.gov was searched on 6 August 2026 using melatonin as the intervention combined separately with osteoarthritis, cartilage, osteoporosis, osteopenia, bone, fracture, sarcopenia, muscle, spinal cord injury, intervertebral-disk degeneration, low-back pain, spine surgery, arthroplasty, orthopedic surgery, sport, athlete, exercise, and recovery. Titles, conditions, assigned interventions, design, recruitment status, outcomes, and results-posting modules were checked. Records in which melatonin was only an endogenous biomarker, a concomitant or exclusion medication, or was replaced by a melatonergic agonist were excluded from the contextual registry map. Registry records were not merged with the bibliographic efficacy flow unless a peer-reviewed primary report independently met the review criteria. The complete registry search specification and accounting are provided in Supplementary File S1, Section S1.3.

### 2.5. Study Selection and Data Extraction

All records were exported with their database source and deduplicated using DOI, PubMed identifier, normalized title, authors, and year. One reviewer (M.B.) screened every title and complete indexed abstract and assessed each potentially eligible report using the information accessible for that report; a second independent screener was not used because the systematic reconstruction occurred during peer review. Complete articles were consulted where accessible. When a complete article was unavailable, assessment was limited to the indexed title, abstract, and bibliographic metadata; such indexed information was not treated as equivalent to full-text assessment. Reports without accessible complete text were retained in the evidence map only to the extent that eligibility and relevant data were ascertainable from indexed information, and all unavailable or unclear items were recorded as not reported and were not imputed. Because complete-article access status was not logged prospectively during the post hoc systematic reconstruction, the number of complete articles accessed among the 437 reports cannot now be reconstructed reliably. No machine-learning classifier or other automation tool was used. The same reviewer (M.B.) extracted data into a predefined evidence-map structure and repeated the complete set of inclusion and extraction decisions in a separate consistency pass, resolving any discrepancy by rechecking the accessible report information. Study investigators were not contacted. Data fields included study design, model or population, sample size when reported, melatonin formulation, dose or concentration, route, timing, duration, comparator, all accessible outcomes compatible with the prespecified domains, principal findings, adverse events, and methodological limitations. Mixed reports were allocated to the highest translational stream represented (human, then animal, then in vitro).

### 2.6. Screen-Level Methodological Limitation Assessment

Methodological limitations were considered separately by evidence stream. The same reviewer (M.B.) applied a screen-level methodological limitation assessment to every included report and repeated it during the consistency pass; no automation tool was used. Limitation flags were recorded from the complete article when accessible and otherwise from the indexed title, abstract, and bibliographic information. The domains were informed by selected elements of SciRAP for in vitro studies [39], SYRCLE for animal studies [40], RoB 2 for randomized human trials, and ROBINS-I for non-randomized comparative studies [41]; however, these instruments were not applied in full. No study-specific overall risk-of-bias judgment or composite score was assigned. The narrative summary below describes selected studies that influenced domain-level conclusions; it is not a complete formal risk-of-bias assessment. The evidence-map flags are interpretive cautions rather than low, unclear, or high risk-of-bias judgments. No formal certainty-of-evidence framework, such as GRADE, was applied; human conclusions were calibrated qualitatively to observed methodological limitations, directness, precision, and consistency, whereas preclinical findings were not assigned clinical certainty ratings.

### 2.7. Evidence Synthesis

Studies were assigned to prespecified synthesis groups by evidence stream (in vitro, animal, or human) and condition: osteoporosis/osteopenia; fracture healing; osteoarthritis; sarcopenia and muscle injury; tendon and ligament disorders; spinal cord injury; intervertebral-disk degeneration and low-back pain; perioperative orthopedic care; and exercise/sports recovery. Eligibility for each synthesis was determined by comparing each report’s population or model, intervention, comparator, and outcomes with the PICOS criteria. Reported relative measures (including risk or hazard ratios) and continuous or absolute measures (including mean differences and within- or between-group changes) were retained with their reported precision when available. No effect estimate was recalculated, converted, or imputed. Results by evidence stream, condition, and design are summarized in Table 2 (mechanisms), Table 3 (exposures), Table 4 (methodological limitations), Table 5 (registered trials), and Supplementary File S1. Statistical pooling was considered only when model or population, indication, formulation, dose, route, comparator, outcome definition, and assessment time were sufficiently comparable; no coherent group met these criteria. A structured qualitative synthesis was therefore performed according to SWiM, considering the direction and magnitude of effect, precision, consistency, directness, and methodological limitations rather than statistical significance alone. Heterogeneity was explored qualitatively across model, population, formulation, dose, route, timing, comparator, and outcome. No subgroup meta-analysis, meta-regression, or sensitivity analysis was performed. Funnel plots and statistical tests for small-study effects were not appropriate; possible missing-result bias was considered by comparing trial registrations with available publications and by noting absent protocols, unposted results, and selective outcome reporting where identifiable. Clinical and preclinical evidence were never pooled, and preclinical findings were treated as hypothesis-generating rather than proof of clinical efficacy.

### 2.8. Study Selection

**Figure 1:**
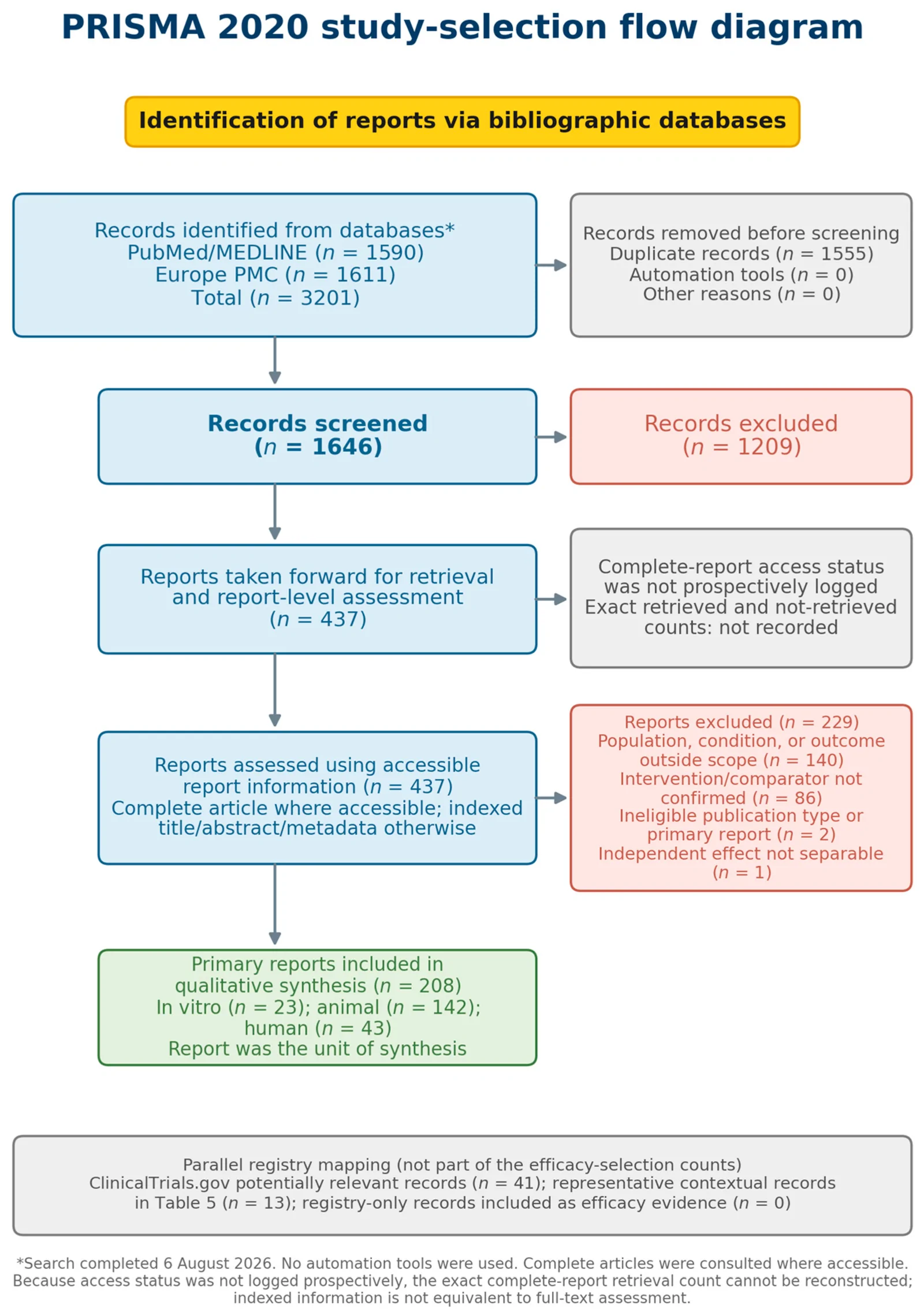
PRISMA 2020 study-selection flow diagram for the bibliographic search completed on 6 August 2026. The report was the unit of synthesis. The 437 reports retained after initial screening were taken forward for retrieval and report-level assessment using complete articles where accessible and indexed information otherwise. Complete-article access status was not logged prospectively; the exact numbers retrieved and not retrieved cannot be reconstructed reliably, and indexed-only assessment is not equivalent to full-text assessment. ClinicalTrials.gov records were mapped in parallel and were not treated as efficacy evidence unless a peer-reviewed report independently met the review criteria. Reporting framework: PRISMA 2020 [37].

The database searches identified 3201 records (PubMed/MEDLINE, 1590; Europe PMC, 1611). After removal of 1555 duplicates, 1646 unique records were screened and 1209 were excluded at title/abstract stage. The remaining 437 potentially eligible reports were taken forward for retrieval and report-level assessment using the information accessible for each report. Complete articles were consulted where accessible; otherwise, assessment was restricted to the indexed title, abstract, and bibliographic metadata. The contemporaneous workflow did not record complete-article access status, so the exact numbers assessed from complete articles and from indexed information alone cannot be reconstructed reliably. The previous statement that no report was lost from retrieval has therefore been removed, and indexed-only assessment is not described as full-text eligibility assessment. On the basis of the available report information, 229 reports were excluded because the population, condition, or outcome was outside scope (*n* = 140), an eligible exogenous melatonin intervention/comparator could not be confirmed (*n* = 86), the publication type did not provide an eligible primary report (*n* = 2), or the independent melatonin effect was not separable (*n* = 1). The qualitative synthesis included 208 primary reports: 23 in vitro, 142 animal, and 43 human. ClinicalTrials.gov yielded 41 potentially relevant records for parallel contextual mapping; 13 representative records are summarized in Table 5, and no registry-only record was counted as efficacy evidence. The bibliographic selection flow is shown in Figure 1.

## 3. Biological Rationale and Molecular Mechanisms

Experimental receptor, cell, and animal studies suggest that melatonin can signal through receptor-dependent and receptor-independent pathways. MT1 and MT2 are G protein-coupled receptors expressed in peripheral tissues, including tissues relevant to the musculoskeletal system. Experimental receptor activation has been associated with changes in cyclic adenosine monophosphate signaling, intracellular calcium handling, inflammatory transcription factors, and differentiation pathways. In bone models, these changes have included osteoblast differentiation and modulation of osteoclast formation through bone morphogenetic proteins, RANKL/osteoprotegerin, and NF-κB pathways [21,22,23,24,25,26,27,28,29,30,31,32,33,34,35,42,43,44,80,81]. These observations establish mechanistic plausibility within the studied systems, not clinical efficacy.

Biochemical and experimental studies describe direct scavenging of reactive oxygen and nitrogen species by melatonin and its metabolites. Its amphiphilic structure permits distribution across cellular compartments, including mitochondria, where experimental work has reported reduced lipid peroxidation and altered electron-transport-chain stability [22,23,24,25,26,27,28]. Nrf2 signaling is another proposed mechanism because it regulates endogenous antioxidant enzymes such as superoxide dismutase, catalase, heme oxygenase-1, and glutathione peroxidase [24,25,26,27,28,29,30,31]. Whether these pathway-level effects occur at clinically achievable musculoskeletal tissue exposures remains uncertain.

In cell and animal models, melatonin has been reported to influence mitochondrial membrane stability, cytochrome c release, apoptosis, mitophagy, NF-κB signaling, and downstream cytokines such as tumor necrosis factor alpha, interleukin 1 beta, and interleukin 6 [28,29,30,31,32,33,34,35]. These outcomes are biologically relevant because chondrocytes, osteoblasts, myocytes, and nucleus pulposus cells are vulnerable to energetic failure and oxidative injury. However, pathway modulation and biomarker improvement cannot be assumed to predict pain relief, functional recovery, or structural disease modification in patients.

**Figure 2:**
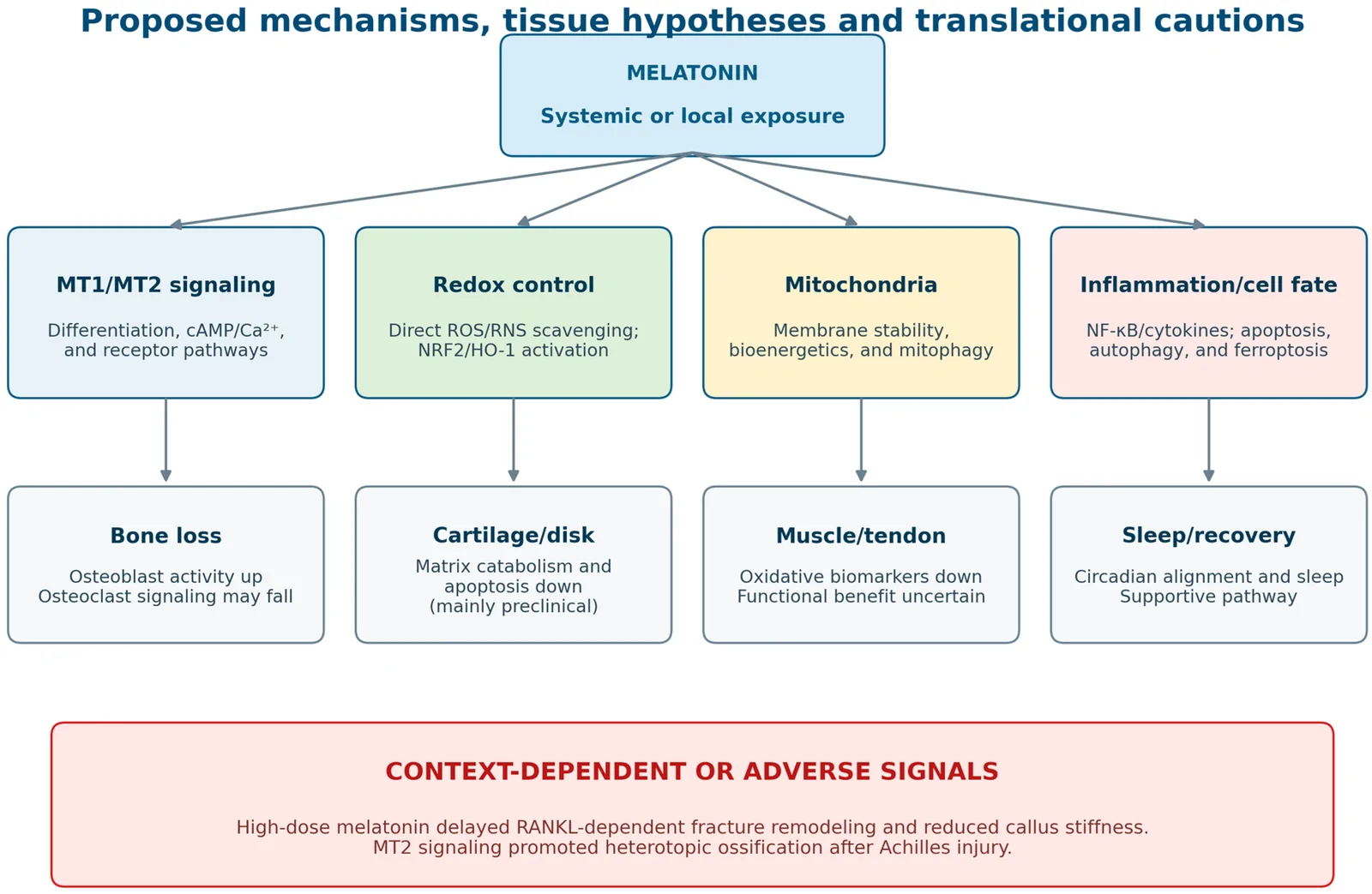
Proposed biological pathways linking melatonin to musculoskeletal outcomes. The lower panel emphasizes that mechanistic plausibility is context-dependent and that adverse preclinical signals have been reported. The adverse signals concern impaired fracture remodeling [63] and MT2-dependent heterotopic ossification after Achilles injury [45].

Emerging work has also explored the relationship between melatonin and ferroptosis, a regulated form of cell death driven by iron-dependent lipid peroxidation. Ferroptosis has been implicated in bone loss and intervertebral-disk degeneration, and melatonin has attenuated this pathway in selected experimental models [29,30,31,32,33,34,35,51]. These effects remain model-, exposure-, and timing-dependent. The proposed pathways and important counter-signals are summarized in Figure 2 and Table 2.

Exposure was reported in non-equivalent units across cell, animal, and human studies. Table 3 therefore records representative concentrations, doses, routes, timing, and durations rather than implying a direct conversion between experimental systems. Parent melatonin was the assigned intervention in these examples; receptor antagonists are identified separately when they were used only as mechanistic probes.

## 4. Osteoporosis and Osteopenia

Bone remodeling depends on the balance between osteoblast-mediated formation and osteoclast-mediated resorption. Controlled cell and animal studies suggest that melatonin can promote osteogenic differentiation and suppress osteoclastogenesis through bone morphogenetic protein, RANKL/osteoprotegerin, NF-κB, and redox pathways [42,43,44,51,62,67,80,81,82,83]. In ovariectomized rats, Guan et al. used 100 mg/kg/day for eight weeks and reported improved bone mass, trabecular microarchitecture, and osteogenic markers [67]. However, abrupt experimental estrogen withdrawal, high exposure, and differences in animal age and timing limit direct translation to postmenopausal osteoporosis. This mechanistic rationale is also discussed in the contextual literature on postmenopausal osteoporosis [84].

Human evidence provides only a preliminary signal. The MOPS pilot randomized 18 perimenopausal women to 3 mg nightly or placebo for six months and found no significant difference in calcaneal bone density or bone-turnover markers [64]. Amstrup et al. randomized 81 postmenopausal women with osteopenia to 1 mg, 3 mg, or placebo for one year; femoral-neck bone mineral density increased dose-dependently, reaching 2.3% in the 3 mg group, while most other sites and turnover markers were unchanged [65]. The MOTS trial combined melatonin with strontium, vitamin D3, and vitamin K2, preventing attribution of any effect to melatonin alone [68]. In the 40-participant MelaOstrong pilot, combined osteogenic loading and melatonin produced within-group lumbar-spine signals but no significant between-group bone-density effect [69].

These trials were small and centered on surrogate outcomes; none was designed or powered to determine fragility-fracture incidence. The Nurses’ Health Study association between night-shift work and fracture risk did not measure melatonin or test supplementation and therefore cannot support treatment efficacy [57]. The certainty of human evidence is low, and melatonin cannot presently be recommended as a substitute for established antiresorptive or anabolic osteoporosis therapy.

## 5. Fracture Healing and Bone Regeneration

Fracture-healing studies are heterogeneous in model, route, and direction of effect. Halici et al. reported more advanced histological union and lower oxidative markers after 30 mg/kg/day intraperitoneal melatonin in rats, but did not test biomechanical competence [62]. Tanrisever et al. found greater histomorphometric bone formation after local dosing with 1.2 or 3 mg in 30 rats, without a dose–response relationship or load-to-failure testing [83]. Conversely, Histing et al. reported reduced callus stiffness, delayed remodeling, lower RANKL expression, and fewer osteoclasts after 50 mg/kg/day [63]. These discordant findings show that increased osteogenesis does not necessarily produce mechanically competent union and that osteoclast activity remains necessary during remodeling. No human trial has demonstrated shorter time to union, fewer non-unions or reoperations, or better patient function; clinical use for fracture healing is therefore unsupported.

## 6. Osteoarthritis

Osteoarthritis is characterized by cartilage degradation, subchondral remodeling, synovial inflammation, and pain. Controlled chondrocyte and surgically induced rodent studies report effects on autophagy, apoptosis, matrix turnover, and inflammatory signaling [47,52,54,70,85,86,87,88,89,90]. Chen et al. combined oxidant-stressed chondrocytes with a destabilization-of-the-medial-meniscus model and linked the response to AMPK/Foxo3 signaling [47]; Zhang et al. and Zhou et al. used pathway interference to support roles for microRNA-140 and the microRNA-146a/NRF2/HO-1 axis [52,86]. These experiments strengthen biological plausibility, but rapidly induced post-traumatic models do not reproduce chronic, metabolically heterogeneous human osteoarthritis.

The only direct human signal identified in the updated literature is hypothesis-generating. Li et al. compared 813 melatonin initiators with 8135 benzodiazepine initiators in a primary-care cohort and observed fewer subsequent analgesic prescriptions and a lower adjusted rate of knee/hip replacement (hazard ratio 0.47, 95% CI 0.30–0.73) [71]. Exposure was not randomized; residual confounding and confounding by indication remain plausible, prescription counts and arthroplasty are indirect outcomes, and the accompanying rat glycine mechanism does not establish causality in humans.

One published rat report frequently cited in this field requires particular caution: its Methods state that the intervention group received a melatonin antagonist even though the title and conclusion interpret the findings as a melatonin effect [20]. Because intervention identity is internally inconsistent, that report was not used as affirmative efficacy evidence. The registered 340-participant randomized knee-osteoarthritis trial (NCT06012175) was completed, but no results were posted as of 6 August 2026. Thus, melatonin remains an investigational analgesic or disease-modifying candidate rather than an established osteoarthritis treatment.

Local delivery may address the short and variable systemic exposure of oral melatonin. Nanoparticles, scaffolds, and sustained-release systems have improved tissue residence or histological cartilage outcomes in experimental models [70,88,89,90]. However, one nanoparticle study used only five mice per group, and human pharmacokinetics, biodegradation, immunogenicity, sterility, intra-articular tolerability, and dose release remain undefined [70]. Delivery innovation therefore does not yet overcome the absence of patient-centered clinical evidence.

At present, melatonin should not replace weight management, exercise therapy, evidence-based analgesia, indicated intra-articular treatment, or surgery. Any use for a coexisting sleep or circadian complaint should be framed explicitly as symptom-directed sleep care rather than treatment of cartilage disease.

## 7. Sarcopenia and Skeletal-Muscle Injury

Skeletal muscle is sensitive to oxidative and inflammatory stress during trauma, sepsis, immobilization, aging, and intensive exercise [91]. These are biologically distinct states and should not be treated as a single therapeutic literature. In a blunt-trauma model, Ostjen et al. studied 28 rats and found that 20 mg/kg/day intraperitoneal melatonin reduced lipid peroxidation and TNF-α/NF-κB-associated inflammation after seven days [48]. Yao et al. reported attenuation of muscle wasting through PI3K/Akt signaling in experimental sepsis [92]. Both are short-term preclinical studies with tissue-level surrogate outcomes and do not establish efficacy in age-related sarcopenia, postoperative atrophy, or rehabilitation.

The main randomized sarcopenia study enrolled 159 older adults into placebo, melatonin 1 mg/day, essential amino acids, or combined treatment for four weeks [66]. The combination increased fat-free mass versus placebo and melatonin alone, but not versus amino acids; strength and inflammation did not improve, and melatonin alone reduced albumin. The short duration and factorial co-intervention prevent a claim that melatonin independently modifies sarcopenia. Longer studies should assess appendicular lean mass together with handgrip, gait speed, SPPB, falls, and independence.

Accordingly, biochemical protection in an acute injury model cannot be extrapolated to durable gains in muscle strength, mobility, or activities of daily living. Current human evidence does not support melatonin as treatment for sarcopenia or postoperative muscle loss, and it should not replace resistance exercise, adequate protein intake, treatment of reversible contributors, or structured rehabilitation.

## 8. Tendinopathy and Ligament Injury

Oxidative stress is relevant to tendon injury and repair [93]. Evidence in tendons and ligaments is exclusively preclinical and not uniformly beneficial. In rat supraspinatus overuse, 10 mg/kg twice-daily intraperitoneal melatonin reduced oxidative and inflammatory measures, but tendon mechanical strength, pain, and function were not assessed [50]. Diabetic-ligament and ovariectomized-Achilles models reported histological or antioxidant changes without consistent functional or biomechanical validation [72,94]. Most importantly, daily melatonin increased heterotopic bone volume and calcified area after Achilles injury through MT2-dependent endothelial-mesenchymal transition [45]. This potential adverse signal precludes generic claims of improved tendon healing. Human trials must assess pain, validated function, imaging, mechanical failure or rerupture, return to activity, and adverse ossification before clinical use can be considered.

## 9. Perioperative Orthopedic Care and Rehabilitation

Perioperative orthopedic studies have evaluated sleep, pain, opioid exposure, or delirium rather than tissue regeneration. Pilot and larger randomized trials after total knee or joint arthroplasty generally found no sustained improvement in validated sleep-quality scores or patient-reported musculoskeletal outcomes [75,76,77,78,79], although some cohorts showed a small increase in sleep duration during the first postoperative nights. These results do not establish enhanced implant integration, wound healing, or functional recovery. Heterogeneous anesthesia, analgesia, rehabilitation, melatonin timing, and sleep measurement further limit comparison. Routine orthopedic recovery protocols are not justified; individualized use may be considered only for a defined sleep/circadian indication and with attention to sedation and fall risk.

## 10. Spinal Cord Injury (Separate Spine-Related Domain)

Spinal cord injury is not an osteoarticular disease and is not classified as one in this review. It was retained as a separate neurological spine-related domain because immobilization-related bone loss, muscle atrophy, pain, and rehabilitation needs overlap with orthopedic practice. Rodent spinal-cord-injury studies report changes in locomotor scores, neuroinflammation, apoptosis, and MT2/NRF2/Keap1 or SIRT1/Drp1 signaling after melatonin [73,74,95]. Reviews and adjacent neurological or mitochondrial literature [96,97,98,99,100,101,102,103,104] provide context rather than direct efficacy evidence in spinal cord injury. These mechanistic findings arise from young-animal models and do not establish human neurological recovery. No human interventional trial of melatonin for functional recovery after spinal cord injury was identified; NCT01741389 examined circadian hemostatic factors in 12 participants with tetraplegia, not neurological treatment. The section is therefore deliberately brief, reported separately from bone and joint evidence, and interpreted as hypothesis-generating only.

## 11. Intervertebral-Disk Degeneration and Low-Back Pain

Intervertebral-disk studies combine cytokine- or oxidant-challenged nucleus pulposus/annulus cells with needle-puncture rat-tail models and report extracellular-matrix preservation, reduced apoptosis or ferroptosis, and improved histology or imaging grades [28,32,35,46,53,56,105,106,107]. These models are useful for target validation but do not reproduce the slow, mechanically driven, and pain-discordant course of human disk degeneration. They do not establish improvement in pain, disability, or quality of life.

The completed MOCHA trial (NCT06476392) and recruiting NCT06859957 evaluate six-week pain intensity in chronic low-back pain, while NCT07344467 evaluates short-term pain after spine surgery. Even if positive, these studies would support an analgesic or perioperative role, not disk regeneration. Future studies must distinguish nonspecific low-back pain from imaging-defined discogenic disease and should pair pain/disability outcomes with structural measures without assuming that imaging change predicts symptom change. Reports on mesenchymal-cell injections and ozone therapy concern other interventions and are cited only for context [108,109].

## 12. Exercise-Induced Muscle Damage and Sports Recovery

Human sports studies are more direct than the spine literature but remain small and heterogeneous. Acute studies in young male athletes reported reductions in lipid-peroxidation, cytokine, or muscle-damage biomarkers after pre-exercise melatonin [49,110]. Ochoa et al. studied 20 trained men undergoing a 50 km mountain run, while Farjallah et al. used a double-blind crossover design in 13 young male footballers and administered 6 mg before a maximal running test [49,58]. The latter study reported selected biochemical effects but no improvement in exercise performance. These samples are small, male-only, short-term, and include multiple outcomes.

Biochemical changes have not consistently translated into strength, speed, injury prevention, or return-to-play benefit [111,112]. The study cited as reference [113] is a rodent swimming model, not a human athlete trial. References [59,60,61] likewise cannot be presented as supplementation-efficacy trials because they concern endogenous secretion, observational sleep/hormonal relationships, or sleep deprivation. Dose, bedtime versus pre-exercise timing, exercise modality, and outcome selection may explain discordance, but current evidence is insufficient to define an ergogenic or injury-prevention effect.

Melatonin should not be promoted as a performance-enhancing agent. If used for sleep or travel-related circadian disruption, clinicians should distinguish that target from tissue recovery, avoid timing that produces daytime somnolence, and consider current sport-governing and local regulatory requirements. Trials should enroll women and men, control training load, diet, and sleep opportunity, and prioritize prespecified performance, injury-incidence, and return-to-play outcomes over isolated biomarkers.

## 13. Formulations, Exposure, Delivery, and Safety

### 13.1. Chemical Identity and Formulation

The therapeutic intervention reviewed here is parent melatonin (N-acetyl-5-methoxytryptamine). Immediate-release and prolonged-release preparations deliver the same molecule but produce different concentration-time profiles. Synthetic melatonin and phytomelatonin likewise contain the same active molecule; plant-derived preparations may contain additional constituents, and clinical superiority over purified synthetic melatonin has not been established [114,115,116]. Chemically distinct melatonergic drugs, including ramelteon, tasimelteon, and agomelatine, were outside the eligibility criteria and are not interchangeable with melatonin [117]. Luzindole and 4-P-PDOT were retained only when used as receptor antagonists to test mechanism, not as therapeutic analogs.

### 13.2. Dose, Concentration, Route, and Timing

The included studies used micromolar cell-culture concentrations, high systemic doses in rodents, low-milligram oral doses in humans, and local or carrier-mediated delivery (Table 3). These exposures are not dose-equivalent. Oral melatonin has variable bioavailability and a short half-life because of first-pass metabolism [118,119], whereas intraperitoneal administration bypasses part of the oral absorption and first-pass process. Accordingly, an effective concentration in a cell system or a dose expressed in mg/kg in a rodent cannot be converted directly into a safe or effective human oral regimen. Clock time, fed state, immediate- versus prolonged-release kinetics, treatment duration, and timing relative to the inflammatory, reparative, and remodeling phases may also change the biological effect.

Nanocarriers, scaffolds, injectable hydrogels, and sustained-release platforms generally carry parent melatonin rather than a synthetic receptor analog and seek to improve local residence or controlled release [120,121,122]. They remain predominantly preclinical. Local delivery may reduce systemic variability but introduces distinct questions about formulation purity, release kinetics, tissue exposure, biodegradation, sterility, immunogenicity, biomaterial toxicity, and compatibility with fixation materials or rehabilitation loads. Histological improvement in a small animal model cannot establish pain relief, functional benefit, or long-term device safety. Product content and purity also vary among supplements; clinical research should therefore use chemically characterized and reproducible formulations. An experimental melatonin carbon-dot composite hydrogel is cited as contextual delivery-platform research, rather than as evidence of human efficacy [123].

### 13.3. Adverse Effects and Context-Dependent Harm

Oral melatonin is generally well tolerated in adult sleep studies, with daytime sleepiness, dizziness, headache, and altered sleep timing among commonly reported adverse effects [124,125,126]. The broader literature does not establish long-term safety at the higher systemic exposures used in musculoskeletal animal experiments or after local implantation. Older and postoperative orthopedic patients may be especially susceptible to somnolence, impaired balance, falls, and additive effects with sedatives; possible interactions with anticoagulant or antiplatelet therapy also require attention. Age, pregnancy, hepatic metabolism, comorbidity, driving or machinery use, concomitant medication, formulation quality, and timing should be considered [126,127,128]. Adverse-event ascertainment was inconsistent across the included musculoskeletal studies, so absence of reported harm cannot be interpreted as evidence of safety.

The efficacy literature also contains negative and potentially adverse signals. High-dose melatonin impaired RANKL-dependent callus remodeling and reduced callus stiffness in a mouse fracture model [63], and MT2-dependent heterotopic ossification increased after Achilles injury in rats [45]. In the four-week sarcopenia trial, the melatonin-alone arm did not improve strength and showed a reduction in serum albumin [66]. Randomized arthroplasty trials reported null or mixed sleep and patient-reported outcomes [75,76,77,78,79]. These findings do not prove comparable harm in patients, but they refute an exclusively beneficial interpretation and show why tissue, healing phase, dose, route, and outcome must be evaluated separately.

## 14. Limitations and Future Directions

### 14.1. Evidence Limitations and Translational Gap

**Figure 3:**
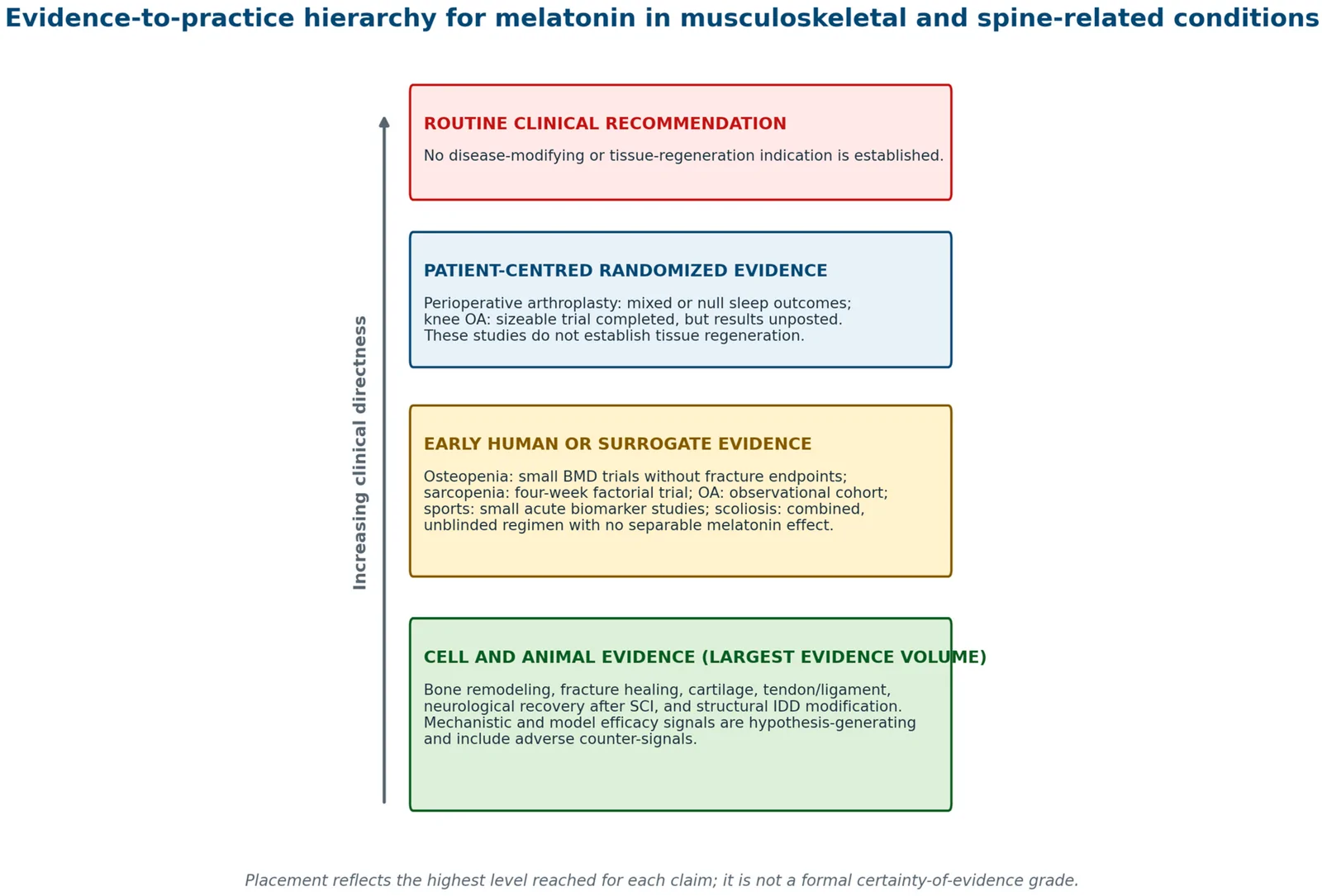
Evidence-to-practice hierarchy for melatonin in the reviewed musculoskeletal and spine-related conditions. Placement reflects the highest level reached for each claim and is not a formal certainty-of-evidence grade. No disease-modifying or tissue-regeneration indication has reached routine clinical recommendation.

The central finding is a pronounced evidence-to-practice gap. The biological rationale is supported by numerous cell and animal experiments, but clinical validation is sparse and indication-specific. Preclinical studies frequently use young animals, acute injury models, high doses, short follow-up, tissue biomarkers, and incomplete reporting of randomization or blinded assessment. Histological cartilage scores, callus area, cytokines, or oxidative markers cannot be assumed to predict pain relief, mechanically competent union, neurological recovery, independence, or avoidance of surgery. Figure 3 separates the evidence streams by their clinical directness so that mechanistic studies, small human surrogate studies, and randomized patient-centered evidence are not interpreted as equivalent.

Pharmacological heterogeneity adds uncertainty. Oral, intraperitoneal, intra-articular, scaffold-based, and nanoparticle-mediated exposure produce different tissue concentrations; time of administration relative to circadian phase and to the inflammatory, proliferative, or remodeling stage may change the effect. The negative fracture-remodeling study [63] and heterotopic-ossification signal [45] show that anti-inflammatory or osteogenic activity is not automatically beneficial at every stage or tissue site. Although the revised article now uses systematic methods, the protocol and eligibility framework were reconstructed post hoc from an original narrative review. Selection and interpretation therefore remain susceptible to reviewer subjectivity. Additional limitations are the lack of prospective protocol registration, single-reviewer screening with a repeated consistency pass rather than duplicate independent review, and non-uniform access to complete reports. Complete-article access status was not logged prospectively during the post hoc systematic reconstruction; therefore, although complete articles were consulted where accessible, the exact number accessed among the 437 reports cannot be reconstructed reliably. Reports lacking accessible complete text were evaluated only from indexed title, abstract, and bibliographic information, which is not equivalent to full-text assessment. The screen-level methodological limitation assessment cannot be interpreted as, or substitute for, a complete study-specific risk-of-bias assessment conducted in duplicate.

### 14.2. Registered Clinical Trials

The ClinicalTrials.gov landscape reinforces the translational gap. A completed, adequately sized knee-osteoarthritis trial is registered, but results have not been posted. Human bone studies remain centered on osteopenia and bone-density surrogates, and no registered trial was identified in which fracture union was the therapeutic target. The sarcopenia evidence includes one four-week trial. No human trial was identified that tested melatonin for neurological recovery after spinal cord injury or structural modification of intervertebral-disk degeneration. Most orthopedic surgical trials target sleep, opioid use, or delirium rather than implant integration or tissue healing. Selected records and their exact status on 6 August 2026 are summarized in Table 5.

### 14.3. Condition-Specific Research Priorities

Future work should progress by indication. Osteoarthritis trials should distinguish analgesia from structural modification, use validated pain/function measures, and assess sleep and chronotype as potential mediators. Osteoporosis studies should test stand-alone melatonin in addition to standard care and ultimately be powered for fragility fractures rather than BMD alone. Fracture-healing studies should prespecify time to clinical/radiographic union, CT bridging where appropriate, weight-bearing progression, reoperation, and patient-reported function, with timing aligned to inflammatory, reparative, and remodeling phases. Sarcopenia studies require 6–12 months of follow-up, standardized exercise/protein exposure, and lean mass, strength, gait speed, SPPB, falls, and independence outcomes.

For spinal cord injury and disk degeneration, exposure-matched dose finding and pharmacokinetic work should precede efficacy trials, and large-animal studies should use clinically realistic timing and delivery. Human SCI trials should stratify by neurological level and AIS grade and use ISNCSCI and SCIM outcomes. Disk studies must separate nonspecific low-back pain from imaging-defined disease. Perioperative trials should standardize anesthesia, analgesia, and rehabilitation and assess actigraphy-defined sleep, delirium, opioid exposure, mobilization, and function. Sports trials should enroll women and men, control training load, diet, and sleep opportunity, and prioritize performance and injury outcomes. Across all domains, formulation, purity, bioavailability, clock time, adherence, adverse events, and concomitant sedatives should be reported, with prospective registration and results posting irrespective of direction.

### 14.4. Guidance for Clinicians

Current evidence does not justify prescribing melatonin as a disease-modifying treatment for osteoarthritis, osteoporosis, sarcopenia, tendinopathy, fracture healing, spinal cord injury, or intervertebral-disk degeneration. The human bone signal concerns BMD rather than fractures, and the disease-specific knee-OA randomized trial has not reported results. Melatonin must not replace exercise therapy, weight management, established anti-osteoporotic medication, fracture stabilization, rehabilitation, or indicated surgery.

If melatonin is used for a recognized sleep or circadian complaint in a patient with a musculoskeletal disorder, the target should be stated explicitly as sleep/circadian symptoms and management should follow applicable guidance. In older or postoperative patients, clinicians should consider somnolence, dizziness, fall risk, driving or machinery use, sedative co-medication, anticoagulant/antiplatelet therapy, comorbidity, and product quality. Athletes should not be told that melatonin is performance-enhancing.

## 15. Conclusions

Experimental studies provide a coherent biological rationale for investigating melatonin in selected musculoskeletal and spine-related conditions, but mechanistic plausibility is not clinical proof. The evidence remains dominated by cell and animal models, and the limited human literature has not established a disease-modifying indication, neurological-recovery effect, formulation, dose, or route for routine orthopedic practice. The practical take-home message is therefore to separate experimental promise from clinical evidence: melatonin may be considered for an independently recognized sleep or circadian problem when appropriate, but it should not be presented as treatment of the underlying musculoskeletal or neurological disorder.

## Figures and Tables

**Table 1:** PICOS framework and prespecified eligibility criteria.

PICOS Element	Prespecified Criterion
Population	Musculoskeletal or spine-related cells and tissues; animals with relevant disease, injury, degeneration, or repair models; and humans with musculoskeletal disorders, orthopedic injuries or procedures, spine-related conditions, rehabilitation needs, sarcopenia, or defined exercise-induced tissue stress. Spinal cord injury was treated as a separate neurological spine-related domain because of its musculoskeletal and orthopedic-rehabilitation consequences, not as an osteoarticular disease.
Intervention	Exogenous melatonin administered alone by any systemic or local route or incorporated into a delivery platform. Multicomponent interventions were eligible only when the independent contribution of melatonin could be estimated; otherwise, they were discussed as indirect evidence.
Comparator	Vehicle or untreated controls in vitro; vehicle, sham, or no-melatonin controls in animals; and placebo, no treatment, usual care, or an active comparator in humans.
Outcomes	Cell viability and differentiation; oxidative, inflammatory, mitochondrial, apoptotic, autophagic, ferroptotic, and extracellular-matrix outcomes; bone architecture, callus formation, histology, biomechanics, and tissue healing; and human pain, function, strength, sleep, recovery, bone density, fracture healing, complications, and adverse events.
Study designs	Controlled primary in vitro experiments, controlled animal studies, randomized clinical trials, and non-randomized comparative human studies.

**Table 2:** Proposed mechanisms, cited biological actions, and critical assessment of the supporting musculoskeletal evidence.

Mechanism	Biological Action	Representative Evidence	Critical Assessment
MT1/MT2 signaling	Experimental studies report receptor-dependent changes in differentiation, inflammatory, and metabolic pathways [42,43,44,45,46].	Bone, disk, and tendon models.	Pharmacological inhibition supports receptor involvement, but the direction is context-dependent: MT2 signaling promoted heterotopic ossification after Achilles injury [45]. Human target-engagement evidence is absent.
Direct antioxidant activity	Biochemical and experimental studies report reactive oxygen/nitrogen species scavenging and less lipid peroxidation [47,48,49,50].	Chondrocyte, muscle-trauma, exercise, and tendon models.	Effects are demonstrated mainly through biochemical or tissue markers. Lower oxidative markers have not been validated as a surrogate for clinical healing or function.
NRF2/HO-1 activation	Experimental models report activation of endogenous antioxidant defenses and possible limitation of ferroptosis [51,52].	Diabetic osteoporosis and OA models.	Pathway manipulation strengthens biological plausibility, but evidence remains limited to cellular and animal models and often uses exposures that cannot be translated directly to patients.
Mitochondrial homeostasis	Experimental models report changes in membrane integrity, bioenergetics, mitophagy, and apoptosis [28,47,53].	Chondrocyte and intervertebral-disk models.	Experimental results are reasonably consistent, but effective human tissue concentrations, pharmacokinetics, and feasible delivery are unknown.
NF-κB/cytokine modulation	Preclinical studies report lower inflammatory transcription and cytokines, including TNF-α, IL-1β, and IL-6 [35,42,48,54,55].	Bone, OA, muscle-trauma, and disk models.	Repeatedly reported preclinically; symptomatic, structural, and functional benefits have not been established in clinical musculoskeletal populations.
Autophagy and ferroptosis	Experimental studies report changes in cellular quality control and iron-dependent lipid peroxidation [32,47,51,56].	Osteoporosis, OA, and disk models.	Promising, but disease-, dose-, and timing-dependent. No validated clinical biomarker or treatment protocol exists.
Chronobiotic effects	Human physiological and sleep studies associate melatonin with circadian alignment and sleep timing/continuity [17,18,19,49,57,58,59,60,61].	Human sleep, exercise, and the observational circadian literature.	Potentially relevant to recovery, but night-shift and endogenous-secretion associations do not demonstrate structural tissue benefit from melatonin treatment.

**Table 3:** Representative melatonin formulations, concentrations, doses, routes, and exposure schedules in the included literature.

Study/Context	Material/Formulation	Concentration or Dose	Route/Timing/Duration	Translational Interpretation
Ma et al. [51]; high-glucose osteoblasts	Parent melatonin	1, 10, or 100 µM	Cell culture; 48 h	In vitro concentration cannot be converted directly to an oral human dose.
Chen et al. [47]; oxidant-stressed chondrocytes	Parent melatonin	10–50 µM	2 h pretreatment; outcomes at 24–72 h	Short, controlled cell exposure does not reproduce chronic human OA.
Chen et al. [47]; DMM rats	Parent melatonin in saline	15 or 30 mg/kg	Intraperitoneal every other day; 8 weeks	High systemic exposure in a surgically induced OA model.
Halici et al. [62]; rat tibial fracture	Parent melatonin	30 mg/kg/day	Intraperitoneal	High systemic exposure; histology was not accompanied by biomechanical testing.
Histing et al. [63]; mouse femoral fracture	Parent melatonin	50 mg/kg/day	Intraperitoneal	Delayed remodeling and reduced callus stiffness provide a potential-harm signal.
Kocadal et al. [50]; rat supraspinatus overuse	Parent melatonin	10 mg/kg twice daily	Intraperitoneal; 3 or 6 weeks	No tendon mechanics, pain, or functional endpoint.
Zhang et al. [45]; rat Achilles injury	Parent melatonin; 4-P-PDOT as MT2 antagonist	50 µg/kg/day; antagonist 1 mg/kg/day	Intraperitoneal; 12 weeks	Melatonin promoted heterotopic ossification; the antagonist was a mechanistic tool, not a melatonin analog.
MOPS [64]; perimenopausal women	Oral parent melatonin	3 mg nightly	Oral; 6 months	No significant bone-density or turnover-marker difference.
Amstrup et al. [65]; osteopenia	Oral parent melatonin	1 or 3 mg nightly	Oral; 12 months	Femoral-neck BMD signal only; no fracture endpoint.
Rondanelli et al. [66]; sarcopenia	Oral parent melatonin	1 mg/day	Oral, 30 min before sleep; 4 weeks	No independent strength benefit; albumin fell in the melatonin-alone group.
Farjallah et al. [58]; exhaustive running	Oral parent melatonin	6 mg	Single pre-exercise dose; acute crossover	Selected biochemical changes occurred without improved exercise performance.

**Table 4:** Detailed methodological limitation summary for selected studies. Design, population, and limitations are shown explicitly; rows from different evidence streams should not be interpreted as equivalent levels of proof. This selective narrative summary is not a complete formal study-specific risk-of-bias assessment.

Condition/Study	Design/Model	Intervention	Principal Finding	Limitations/Clinical Applicability
Osteoporosis; Guan et al. [67]	OVX rats	100 mg/kg/day, 8 weeks	Improved bone mass, microarchitecture, and osteogenic markers.	Abrupt OVX model, very high dose, no fracture or human outcome.
Osteopenia; MOPS [64]	Pilot double-blind RCT; *n* = 18	3 mg nightly vs. placebo, 6 months	No significant bone-density or turnover-marker difference; exploratory remodeling/QoL signal.	Very small, imbalanced groups; short duration; no fracture endpoint.
Osteopenia; Amstrup et al. [65]	Double-blind RCT; *n* = 81	1 mg, 3 mg, or placebo, 1 year	Dose-related femoral-neck BMD increase; 2.3% at 3 mg.	Modest surrogate effect; most sites unchanged; no fracture endpoint.
Osteopenia; MOTS [68]	Double-blind RCT; *n* = 22	Melatonin 5 mg plus strontium, D3, K2 vs. placebo	Lumbar-spine and femoral-neck BMD increased.	Combination prevents attribution to melatonin; tiny trial; surrogate outcomes.
Osteopenia; MelaOstrong [69]	Pilot 2 × 2 RCT; *n* = 40	5 mg/placebo × osteogenic loading/mock, 1 year	Within-group lumbar BMD signal with combined intervention.	No significant between-group BMD effect; small factorial study; no fractures.
Fracture; Halici et al. [62]	Rat tibial fracture; *n* = 80	30 mg/kg/day intraperitoneally	Improved histological union and oxidative markers.	No biomechanical testing; high systemic dose; animal model.
Fracture; Histing et al. [63]	Mouse femoral fracture; 30 treated/30 controls	50 mg/kg/day intraperitoneally	Reduced callus stiffness and delayed RANKL-dependent remodeling.	High-dose model but important contradictory/potential-harm evidence.
OA; Chen et al. [47]	Chondrocytes and DMM rats	10–50 µM in vitro; 15/30 mg/kg intraperitoneally every other day in vivo	Improved autophagy, redox balance, and cartilage histology.	High rodent exposure; surgical OA; no pain or function.
OA; Liang et al. [70]	ACLT mice; *n* = 5/group	Targeted nanoparticles every 2 weeks	Improved targeting and OARSI histology.	Very small groups; no human PK, safety, pain, or function.
OA; Li et al. [71]	Observational cohort: 813 vs. 8135; rat model	Melatonin initiators vs. benzodiazepine initiators	Fewer analgesic prescriptions; arthroplasty HR 0.47.	Nonrandomized, indirect outcomes, residual confounding and confounding by indication; animal mechanism does not prove human causality.
Sarcopenia; Rondanelli et al. [66]	Four-arm RCT; *n* = 159	1 mg, amino acids, combination, or placebo; 4 weeks	Combination increased fat-free mass vs. placebo/melatonin, not vs. amino acids; no strength benefit.	Short duration; independent melatonin effect not established; albumin fell with melatonin alone.
Muscle trauma; Ostjen et al. [48]	Rat blunt-trauma model; *n* = 28	20 mg/kg/day, 7 days	Reduced oxidative/inflammatory markers.	Approximately 7/group; no strength or functional recovery.
Exercise; Ochoa et al. [49]	Placebo-controlled; *n* = 20 trained men	Peri-exercise oral melatonin; 50 km run	Reduced oxidative and inflammatory biomarkers.	Small acute biomarker study; no injury or clinically meaningful recovery endpoint.
Exercise; Farjallah et al. [58]	Double-blind crossover; *n* = 13 male footballers	6 mg orally before maximal running	Selected damage and oxidative markers improved; exercise performance did not.	Small male-only acute study with multiple outcomes.
Tendinopathy; Kocadal et al. [50]	Rat supraspinatus overuse; *n* = 50	10 mg/kg intraperitoneally twice daily	Reduced oxidative/inflammatory measures.	No tendon mechanics, pain, or function; high systemic dose.
Ligament injury; Adamska et al. [72]	Diabetic rat MCL injury; *n* = 40	3 mg/kg/day, 4 weeks	Improved histology and antioxidant status.	No mechanical-strength or functional testing; model-specific.
Achilles injury; Zhang et al. [45]	Rat tenotomy; *n* = 6/group	50 µg/kg/day intraperitoneally for 12 weeks; 4-P-PDOT 1 mg/kg/day	Increased heterotopic ossification through MT2/EndMT.	Potential adverse signal; 4-P-PDOT was a mechanistic antagonist, not an analog therapy.
SCI; Yan/Zhong et al. [73,74]	Rodent SCI models	Systemic melatonin	Improved locomotor and molecular outcomes via NRF2/Keap1 or SIRT1/Drp1.	Young-animal acute models; no human neurological-recovery trial.
Disk degeneration [28,32,35,56]	Cells and needle-puncture rodent models	Systemic/local experimental dosing	Matrix preservation; less apoptosis/ferroptosis; improved histology.	Does not establish pain, disability, or structural modification in humans.
Scoliosis; Herdea et al. [36]	Prospective randomized case–control study; *n* = 51; unblinded	Oral melatonin 1.5 mg/day plus calcium 600 mg/day and vitamin D 2000 IU/day; 1 year	The combined regimen was associated with less Cobb-angle progression.	Contextual only: odd/even allocation, no investigator blinding, and no separable melatonin effect.
Arthroplasty sleep [75,76,77,78,79]	Pilot and larger randomized trials	Oral melatonin vs. placebo	No consistent sustained benefit in validated sleep quality or patient-reported orthopedic outcomes.	Supportive-care endpoints; heterogeneous protocols; no tissue-healing or implant-integration evidence.

**Table 5:** Selected ClinicalTrials.gov records relevant to musculoskeletal and orthopedic practice (status verified 6 August 2026).

Domain	Trial/Design	Status	Primary Endpoint	Critical Interpretation
Knee OA	NCT06012175; *n* = 340; melatonin 3 mg nightly vs. placebo, 12 weeks	Completed; no results posted	WOMAC pain at week 12	First sizeable disease-specific OA RCT; efficacy cannot be judged, and the trial does not test structural modification.
Osteopenia	NCT01690000; *n* = 81; 1 or 3 mg nightly vs. placebo, 12 months	Completed; results posted	DXA BMD	Small surrogate-outcome trial; doses pooled in registry analysis; no fracture outcome [65].
Osteopenia	NCT04233112; *n* = 40; 2 × 2 melatonin 5 mg/placebo × osteogenic loading/mock	Active, not recruiting; no registry results	DXA BMD at 12 months	Small factorial design limits main-effect and interaction estimates; a publication reports no between-group BMD effect [69].
Postmenopausal osteoporosis	NCT04864509; *n* = 40 estimated; melatonin 10 mg vs. placebo	Unknown status; no results; last updated 29 April 2021	Bone-marrow MSC gene expression	Must not be described as active; mechanistic primary endpoint cannot establish fracture prevention.
Sarcopenia	NCT03784495; *n* = 159; melatonin, amino acids, combination, placebo; 4 weeks	Completed; no registry results	Fat-free mass and handgrip	Exposure is too short for sustained disease modification; published results do not isolate a beneficial melatonin effect [66].
SCI	NCT01741389; *n* = 12; prolonged-release melatonin vs. placebo crossover	Completed; no results posted	Circadian variation in hemostatic factors	Basic-science coagulation study, not neurological recovery; no disease-modifying human SCI trial identified.
Chronic low-back pain	NCT06476392; *n* = 220; melatonin 10 mg nightly vs. placebo, 6 weeks	Completed; no results posted	0–10 pain NRS	Clinically relevant pain trial but does not test disk regeneration.
Chronic low-back pain	NCT06859957; *n* = 240 estimated; 10 mg vs. placebo, 6 weeks	Recruiting; no results	0–10 pain NRS	May clarify short-term analgesia; cannot establish structural disk modification.
THA/TKA	NCT04795336; *n* = 294; melatonin 5 mg vs. placebo, 14 days	Completed; registry results posted	Epworth Sleepiness Score	Sleep-focused; no evidence of implant integration or tissue-healing benefit.
Lower-limb fracture surgery	NCT04335968; *n* = 357; melatonin 4 mg vs. placebo through postoperative day 5	Completed; no results posted	Postoperative delirium	Important supportive-care trial; endpoint is delirium, not fracture union or function.
TKA	NCT04247646; *n* = 120; sublingual melatonin 5 mg vs. placebo for 29 nights	Active, not recruiting; no results	Opioid use through day 28	Supportive-care endpoint; does not assess fixation or tissue healing.
THA	NCT07732725; *n* = 150; 5 mg vs. 10 mg vs. placebo perioperatively and 14 nights	Not yet recruiting; first posted 29 Jul 2026	Sleep quality at day 14	Relevant dose-comparison design but currently provides no evidence.
Exercise recovery	NCT06617351; *n* = 24 estimated; 5 mg three times daily for 72 h vs. placebo	Not yet recruiting; estimated dates elapsed	Multiple sleep, soreness, force, CK, and immune outcomes	Small exploratory study with high outcome multiplicity and uncertain current conduct.

## Data Availability

The original contributions presented in this study are included in the article/Supplementary Material. Further inquiries can be directed to the corresponding author.

## Supplementary Materials

The following supporting information can be downloaded at: https://www.mdpi.com/article/10.3390/medicina62101946/s1, Supplementary File S1 provides the exact PubMed/MEDLINE, Europe PMC, and ClinicalTrials.gov search specifications; database and study-selection accounting; aggregate exclusion reasons; selected reports that might appear eligible but were excluded; the complete 208-report evidence map with design-stream assignments and screen-level methodological limitation assessment flags; and the registration, protocol, and amendment record. Because complete-article access was not logged prospectively, Supplementary File S1 does not provide a reliable report-level full-text access variable, and no such data were imputed. A completed PRISMA 2020 checklist is submitted as a separate Supplementary File.

## Abbreviations

AMPK, adenosine monophosphate-activated protein kinase; BMD, bone mineral density; BMP, bone morphogenetic protein; ECM, extracellular matrix; IDD, intervertebral disk degeneration; IL, interleukin; MT1/MT2, melatonin receptors 1 and 2; NF-κB, nuclear factor kappa B; Nrf2, nuclear factor erythroid 2-related factor 2; OA, osteoarthritis; OPG, osteoprotegerin; RANKL, receptor activator of nuclear factor kappa B ligand; ROS, reactive oxygen species; SCI, spinal cord injury; SIRT1, sirtuin 1; TNF-α, tumor necrosis factor alpha.
