# Supplementary material for: Melatonin in Musculoskeletal and Spine-Related Conditions: A Systematic Review of Preclinical and Clinical Evidence
Source: Medicina (Kaunas). 2026 Oct 9;62(10):1946. doi: 10.3390/medicina62101946 (PMC13707208; doi:10.3390/medicina62101946)
Supplement: Supplementary file 1 [file medicina-62-01946-s001.zip › Supplementary_File_S1_PRISMA_Aligned.pdf]

Supplementary File S1

Exact search strategies, study-selection accounting, and record-level evidence map

Manuscript: Melatonin in Musculoskeletal and Spine-Related Conditions: A Systematic Review of Preclinical and Clinical Evidence
Search date: 6 August 2026 | **Databases:** PubMed/MEDLINE and Europe PMC

S1. Search Strategies

Both databases were searched from inception through 6 August 2026. The searches were intentionally broad across bone, cartilage, muscle, tendon/ligament, spinal cord, intervertebral disc, orthopedic care, and exercise/sports recovery. Publication type and eligibility restrictions were applied during screening rather than in the search string.

PubMed/MEDLINE (n = 1,590)

```
("Melatonin"[Mesh] OR melatonin[tiab] OR "N-acetyl-5-methoxytryptamine"[tiab]) AND (bone[tiab] OR osteoporosis[tiab] OR osteopenia[tiab] OR "fracture healing"[tiab] OR osteoblast*[tiab] OR osteoclast*[tiab] OR cartilage[tiab] OR chondrocyt*[tiab] OR osteoarthrit*[tiab] OR "skeletal muscle"[tiab] OR sarcopeni*[tiab] OR tendon*[tiab] OR ligament*[tiab] OR "spinal cord injury"[tiab] OR "intervertebral disc"[tiab] OR orthop*[tiab] OR arthroplast*[tiab] OR ((exercise[tiab] OR athlete*[tiab] OR sport*[tiab]) AND (supplement*[tiab] OR treat*[tiab] OR administer*[tiab])))
```

Search executed 6 August 2026; records returned: 1,590.

Europe PMC (n = 1,611)

```
TITLE_ABS:melatonin AND (TITLE_ABS:bone OR TITLE_ABS:osteoporosis OR TITLE_ABS:osteopenia OR TITLE_ABS:"fracture healing" OR TITLE_ABS:osteoblast* OR TITLE_ABS:osteoclast* OR TITLE_ABS:cartilage OR TITLE_ABS:chondrocyt* OR TITLE_ABS:osteoarthrit* OR TITLE_ABS:"skeletal muscle" OR TITLE_ABS:sarcopeni* OR TITLE_ABS:tendon* OR TITLE_ABS:ligament* OR TITLE_ABS:"spinal cord injury" OR TITLE_ABS:"intervertebral disc" OR TITLE_ABS:orthop* OR TITLE_ABS:arthroplast* OR ((TITLE_ABS:exercise OR TITLE_ABS:athlete* OR TITLE_ABS:sport*) AND (TITLE_ABS:supplement* OR TITLE_ABS:treat* OR TITLE_ABS:administer*))) AND FIRST_PDATE:[* TO 2026-08-06]
```

Search executed 6 August 2026; records returned: 1,611.

ClinicalTrials.gov (parallel registry mapping; n = 41 potentially relevant records)

ClinicalTrials.gov was searched on 6 August 2026 with melatonin entered as the intervention and each of the following condition or free-text concepts applied separately: osteoarthritis; cartilage; osteoporosis; osteopenia; bone; fracture; sarcopenia; muscle; spinal cord injury; intervertebral disc degeneration; low-back pain; spine surgery; arthroplasty; orthopedic surgery; sport; athlete; exercise; and recovery. Titles, conditions, interventions, study design, recruitment status, primary outcomes, and results-posting modules were checked. Records in which melatonin was only an endogenous biomarker, a concomitant or

exclusion medication, or was replaced by a melatonergic agonist were excluded from the contextual map. Registry-only records were not merged into the bibliographic efficacy flow. Forty-one potentially relevant registry records were identified before screening; 13 representative musculoskeletal or orthopedic records are reported in main-text Table 4, and none was treated as efficacy evidence without an independently eligible peer-reviewed report.

S2. Study-Selection Accounting

Selection stage	n	Operational note
Records identified	3,201	PubMed/MEDLINE 1,590; Europe PMC 1,611
Duplicates removed	1,555	Matched using DOI, PMID, normalized title, authors, and year
Unique records screened	1,646	Title and complete indexed abstract
Excluded at title/abstract stage	1,209	Did not meet the prespecified intervention/domain/design criteria
Reports taken forward for retrieval and report-level assessment	437	Complete articles were consulted where accessible; otherwise indexed title, abstract, and metadata were used.
Complete-article access status	NR	Not recorded prospectively; exact numbers accessed and not accessed cannot be reconstructed reliably.
Reports assessed for eligibility using accessible information	437	Indexed-only assessment was not treated as equivalent to full-text assessment; unavailable items were recorded as not reported.
Excluded after report-level assessment	229	On the basis of accessible report information; aggregate reasons reported below.
Included reports	208	23 in vitro; 142 animal; 43 human

S2.1. Aggregate Reasons for Exclusion after Report-Level Assessment

Reason	n
Wrong population, condition, or outcome	140
Eligible exogenous melatonin intervention/comparator not confirmed	86
Ineligible publication type or no eligible primary report	2
Independent melatonin effect not separable	1
Total	229

S2.2. Screening and Screen-Level Methodological Limitation Assessment

One reviewer performed the post hoc reconstruction during revision and repeated all screening, extraction, and screen-level methodological limitation decisions in a separate consistency pass; no second reviewer or automation was used. Investigators were not contacted, and missing or unclear information was recorded as not reported and was not imputed. Complete articles were consulted where accessible; otherwise eligibility and data ascertainment were limited to indexed title, abstract, and metadata, which was not considered equivalent to full-text assessment. Complete-article access status was not prospectively logged, so exact accessed and not-accessed counts cannot be reconstructed reliably. Mixed reports were assigned to the highest translational stream (human, animal, then in vitro). Selected domains from SciRAP, SYRCLE, RoB 2, and ROBINS-I informed the screen-level flags; the tools were not applied in full, and no study-specific overall risk-of-bias judgment or composite score was assigned.

S2.3. Selected Reports That Might Appear Eligible but Were Excluded

Report	Reason for exclusion
PMID 28579893 - spinal cord injury report	Retracted report; excluded from the evidence synthesis.
PMID 41705311 - spinal cord injury-related report	Expression of concern and bladder-focused endpoint outside the prespecified musculoskeletal efficacy outcomes.
PMID 42333504 - infected bone-defect scaffold	Multicomponent CuHP/near-infrared/melatonin platform; independent melatonin effect not separable.
PMID 41705491 - bone-regeneration nanovesicle	Multifunction combination platform; independent melatonin effect not separable.
PMID 40744145 - osteoarthritis hydrogel	Multi-agent melatonin/ammonia-borane/bioactive-glass system; independent effect not separable.
PMID 40412506 - tendon hydrogel	Sequential melatonin/PDGF-BB intervention; independent melatonin effect not separable.
PMID 42120967 - spinal cord injury nanozyme	Cerium-melatonin nanozyme plus apoptotic vesicles; complex combination without separable melatonin effect.
PMID 41221649 - spinal cord injury hydrogel	Sequential melatonin/ibuprofen platform; independent melatonin effect not separable.

Report	Reason for exclusion
PMID 36213800 - disc-herniation surgery	Melatonin versus zolpidem for short-term postoperative symptoms; no eligible disease-modifying or disc-regeneration outcome.

S3. Record-Level Evidence Map of Included Reports

The table lists all 208 reports included in the qualitative synthesis. The final column provides screen-level methodological limitation flags—interpretive cautions, not formal risk-of-bias judgments—based on accessible report information. Identifier links can be reconstructed from the PMID or DOI. Domain labels are organizational and do not imply equivalence between evidence streams.

ID	Year	Domain / stream	Report and identifier	Design and screen-level methodological limitation assessment
R0028	2026	Bone loss/osteogenesis<br>Animal	Melatonin Counteracts Mechanical Unloading-Induced Bone Loss Through YTHDF3-Mediated m6A Modification of Dapk2 mRNA.<br>Sun Q, Xu L, Li Z, Zhang J, Zhao X, Zhang L, Zhang X, Zhao J, Tan Y, Wang L, Zhang G, Hu Z, Zhang S, Shi F.<br>Journal of pineal research (2026)<br>PMID 42149012; doi:10.1111/jpi.70146	Controlled animal/mixed preclinical study. Screen-level limitation flag: incomplete reporting of randomization, allocation concealment, blinding, attrition, and prospective sample-size justification.
R0033	2026	Bone loss/osteogenesis<br>Animal	Melatonin improves osteogenic differentiation in a high-glucose environment by activating NRF2 to promote autophagy through the regulation of cross-talk between macrophages and bone marrow mesenchymal stem cells.<br>Zhang J, Zhu L, Zhou J, Yu Q, Yang G, Luo C, Meng J, Mao K, Liu J, Mou D, Yang X.<br>Inflammation research : official journal of the European Histamine Research Society ... [et al.] (2026)<br>PMID 42096091; doi:10.1007/s00011-026-02267-w	Controlled animal/mixed preclinical study. Screen-level limitation flag: incomplete reporting of randomization, allocation concealment, blinding, attrition, and prospective sample-size justification.
R0144	2025	Bone loss/osteogenesis<br>Animal	Melatonin inhibits adipogenesis and enhances osteogenesis of BMSCs through increasing KLF5 expression.<br>Wang S, Tian C, Liu J, Feng Z, Zhao D, Yang A, Xiang D, Chen C, Xia Y, Geng B.<br>Cellular signalling (2025)<br>PMID 40701316; doi:10.1016/j.cellsig.2025.112017	Controlled animal/mixed preclinical study. Screen-level limitation flag: incomplete reporting of randomization, allocation concealment, blinding, attrition, and prospective sample-size justification.
R0179	2025	Bone loss/osteogenesis<br>Animal	Melatonin Promotes Osteogenic Differentiation of Rat Adipose-Derived Stem Cells via the p38/MAPK Signaling Pathway.<br>Meng S, Han S, Kong M, Liu Z, Lin A, Qu C, Li L, Ma X, Wang Y.<br>FASEB journal : official publication of the Federation of American Societies for Experimental Biology (2025)<br>PMID 40387393; doi:10.1096/fj.202403193rr	Controlled animal/mixed preclinical study. Screen-level limitation flag: incomplete reporting of randomization, allocation concealment, blinding, attrition, and prospective sample-size justification.
R0201	2025	Bone loss/osteogenesis<br>Animal	Melatonin attenuates inflammatory bone loss by alleviating mitophagy and lactate production.<br>Lin Z, Gu Y, Liu Y, Chen Z, Fang S, Wang Z, Liu Z, Lin Q, Hu Y, Jiang N, Yu B, Liu G.<br>Apoptosis : an international journal on programmed cell death (2025)<br>PMID 40064756; doi:10.1007/s10495-025-02096-y	Controlled animal/mixed preclinical study. Screen-level limitation flag: incomplete reporting of randomization, allocation concealment, blinding, attrition, and prospective sample-size justification.
R0270	2024	Bone loss/osteogenesis<br>Animal	Cartilage defect repair in a rat model via a nanocomposite hydrogel loaded with melatonin-loaded gelatin nanofibers and menstrual blood stem cells: an in vitro and in vivo study.<br>Yuan L, Yao L, Ren X, Chen X, Li X, Xu Y, Jin T.<br>Journal of materials science. Materials in medicine (2024)<br>PMID 39347832; doi:10.1007/s10856-024-06820-z	Controlled animal/mixed preclinical study. Screen-level limitation flag: incomplete reporting of randomization, allocation concealment, blinding, attrition, and prospective sample-size justification.
R0271	2024	Bone loss/osteogenesis<br>Animal	Melatonin Regulates Osteoblast Differentiation through the m6A Reader hnRNPA2B1 under Simulated Microgravity.<br>Sun Q, Xu L, Hu Z, Liu J, Yu T, Li M, Zhang S, Shi F.<br>Current issues in molecular biology (2024)<br>PMID 39329924; doi:10.3390/cimb46090572	Controlled animal/mixed preclinical study. Screen-level limitation flag: incomplete reporting of randomization, allocation concealment, blinding, attrition, and prospective sample-size justification.

ID	Year	Domain / stream	Report and identifier	Design and screen-level methodological limitation assessment
R0286	2024	Bone loss/osteogenesis<br>Animal	Melatonin prevents bone loss in osteoporotic rats with valproic acid treatment by anti-inflammatory and anti-oxidative stress.<br>Tao ZS, Hu XF, Sun T.<br>International immunopharmacology (2024)<br>PMID 39154533; doi:10.1016/j.intimp.2024.112932	Controlled animal/mixed preclinical study. Screen-level limitation flag: incomplete reporting of randomization, allocation concealment, blinding, attrition, and prospective sample-size justification.
R0378	2023	Bone loss/osteogenesis<br>Animal	Melatonin induces RAW264.7 cell apoptosis via the BMAL1/ROS/MAPK-p38 pathway to improve postmenopausal osteoporosis.<br>Wang X, Jiang W, Pan K, Tao L, Zhu Y.<br>Bone & joint research (2023)<br>PMID 37907083; doi:10.1302/2046-3758.1211.bjr-2022-0425.r3	Controlled animal/mixed preclinical study. Screen-level limitation flag: incomplete reporting of randomization, allocation concealment, blinding, attrition, and prospective sample-size justification.
R0409	2023	Bone loss/osteogenesis<br>Animal	[Melatonin promotes osteogenesis of bone marrow mesenchymal stem cells by improving the inflammatory state in ovariectomized rats].<br>Guan H, Cao R, Zhao Y, Zhang J, Li H, Duan X, Li Y, Kong N, Tian R, Wang K, Yang P.<br>Zhongguo xiu fu chong jian wai ke za zhi = Zhongguo xiufu chongjian waike zazhi = Chinese journal of reparative and reconstructive surgery (2023)<br>PMID 37586803; doi:10.7507/1002-1892.202304001	Controlled animal/mixed preclinical study. Screen-level limitation flag: incomplete reporting of randomization, allocation concealment, blinding, attrition, and prospective sample-size justification.
R0430	2023	Bone loss/osteogenesis<br>Animal	Melatonin Promotes BMSCs Osteoblastic Differentiation and Relieves Inflammation by Suppressing the NF-κB Pathways.<br>Hu Y, Xiong Y, Zha K, Tao R, Chen L, Xue H, Yan C, Lin Z, Endo Y, Cao F, Zhou W, Liu G.<br>Stem cells international (2023)<br>PMID 37274021; doi:10.1155/2023/7638842	Controlled animal/mixed preclinical study. Screen-level limitation flag: incomplete reporting of randomization, allocation concealment, blinding, attrition, and prospective sample-size justification.
R0463	2023	Bone loss/osteogenesis<br>Animal	Melatonin activates mitochondrial unfolded protein response to preserve osteogenic potential of senescent BMSCs via upregulating PDI-6.<br>Li F, Lun D, Liu D, Jia Z, Zhu Z, Liu Z, Li X.<br>Biochimie (2023)<br>PMID 36708867; doi:10.1016/j.biochi.2023.01.015	Controlled animal/mixed preclinical study. Screen-level limitation flag: incomplete reporting of randomization, allocation concealment, blinding, attrition, and prospective sample-size justification.
R0488	2023	Bone loss/osteogenesis<br>Animal	Melatonin suppresses bone marrow adiposity in ovariectomized rats by rescuing the imbalance between osteogenesis and adipogenesis through SIRT1 activation.<br>Huang X, Chen W, Gu C, Liu H, Hou M, Qin W, Zhu X, Chen X, Liu T, Yang H, He F.<br>Journal of orthopaedic translation (2023)<br>PMID 36381247; doi:10.1016/j.jot.2022.10.002	Controlled animal/mixed preclinical study. Screen-level limitation flag: incomplete reporting of randomization, allocation concealment, blinding, attrition, and prospective sample-size justification.
R0518	2022	Bone loss/osteogenesis<br>Animal	Melatonin Inhibits the Ferroptosis Pathway in Rat Bone Marrow Mesenchymal Stem Cells by Activating the PI3K/AKT/mTOR Signaling Axis to Attenuate Steroid-Induced Osteoporosis.<br>Li M, Yang N, Hao L, Zhou W, Li L, Liu L, Yang F, Xu L, Yao G, Zhu C, Xu W, Fang S.<br>Oxidative medicine and cellular longevity (2022)<br>PMID 36035224; doi:10.1155/2022/8223737	Controlled animal/mixed preclinical study. Screen-level limitation flag: incomplete reporting of randomization, allocation concealment, blinding, attrition, and prospective sample-size justification.

ID	Year	Domain / stream	Report and identifier	Design and screen-level methodological limitation assessment
R0566	2022	Bone loss/osteogenesis<br>Animal	Melatonin Mediates Osteoblast Proliferation Through the STIM1/ORAI1 Pathway.<br>Cao L, Yang K, Yuan W, Zhou S, Zhao R, Qiu S.<br>Frontiers in pharmacology (2022)<br>PMID 35392575; doi:10.3389/fphar.2022.851663	Controlled animal/mixed preclinical study. Screen-level limitation flag: incomplete reporting of randomization, allocation concealment, blinding, attrition, and prospective sample-size justification.
R0576	2022	Bone loss/osteogenesis<br>Animal	Melatonin enhances osteoblastogenesis of senescent bone marrow stromal cells through NSD2-mediated chromatin remodelling.<br>Xie Y, Han N, Li F, Wang L, Liu G, Hu M, Wang S, Wei X, Guo J, Jiang H, Wang J, Li X, Wang Y, Wang J, Bian X, Zhu Z, Zhang H, Liu C, Liu X, Liu Z.<br>Clinical and translational medicine (2022)<br>PMID 35220680; doi:10.1002/ctm2.746	Controlled animal/mixed preclinical study. Screen-level limitation flag: incomplete reporting of randomization, allocation concealment, blinding, attrition, and prospective sample-size justification.
R0627	2022	Bone loss/osteogenesis<br>Animal	Exogenous melatonin prevents type 1 diabetes mellitus-induced bone loss, probably by inhibiting senescence.<br>Gong Z, Da W, Tian Y, Zhao R, Qiu S, Wu Q, Wen K, Shen L, Zhou R, Tao L, Zhu Y.<br>Osteoporosis international : a journal established as result of cooperation between the European Foundation for Osteoporosis and the National Osteoporosis Foundation of the USA (2022)<br>PMID 34519833; doi:10.1007/s00198-021-06061-8	Controlled animal/mixed preclinical study. Screen-level limitation flag: incomplete reporting of randomization, allocation concealment, blinding, attrition, and prospective sample-size justification.
R0675	2021	Bone loss/osteogenesis<br>Animal	Melatonin Inhibits Osteoclastogenesis and Bone Loss in Ovariectomized Mice by Regulating PRMT1-Mediated Signaling.<br>Choi JH, Jang AR, Park MJ, Kim DI, Park JH.<br>Endocrinology (2021)<br>PMID 33713122; doi:10.1210/endocr/bqab057	Controlled animal/mixed preclinical study. Screen-level limitation flag: incomplete reporting of randomization, allocation concealment, blinding, attrition, and prospective sample-size justification.
R0699	2020	Bone loss/osteogenesis<br>Animal	Melatonin Suppresses Ferroptosis Induced by High Glucose via Activation of the Nrf2/HO-1 Signaling Pathway in Type 2 Diabetic Osteoporosis.<br>Ma H, Wang X, Zhang W, Li H, Zhao W, Sun J, Yang M.<br>Oxidative medicine and cellular longevity (2020)<br>PMID 33343809; doi:10.1155/2020/9067610	Controlled animal/mixed preclinical study. Screen-level limitation flag: incomplete reporting of randomization, allocation concealment, blinding, attrition, and prospective sample-size justification.
R0730	2020	Bone loss/osteogenesis<br>Animal	Melatonin Rescues the Ti Particle-Impaired Osteogenic Potential of Bone Marrow Mesenchymal Stem Cells via the SIRT1/SOD2 Signaling Pathway.<br>Zhang Y, Zhu X, Wang G, Chen L, Yang H, He F, Lin J.<br>Calcified tissue international (2020)<br>PMID 32767062; doi:10.1007/s00223-020-00741-z	Controlled animal/mixed preclinical study. Screen-level limitation flag: incomplete reporting of randomization, allocation concealment, blinding, attrition, and prospective sample-size justification.
R0789	2020	Bone loss/osteogenesis<br>Animal	Rapamycin could increase the effects of melatonin against age-dependent bone loss.<br>Tao ZS, Lu HL, Ma NF, Zhang RT, Li Y, Yang M, Xu HG.<br>Zeitschrift fur Gerontologie und Geriatrie (2020)<br>PMID 31781847; doi:10.1007/s00391-019-01659-4	Controlled animal/mixed preclinical study. Screen-level limitation flag: incomplete reporting of randomization, allocation concealment, blinding, attrition, and prospective sample-size justification.

ID	Year	Domain / stream	Report and identifier	Design and screen-level methodological limitation assessment
R0808	2020	Bone loss/osteogenesis<br>Animal	Melatonin promotes osteoblastic differentiation and regulates PDGF/AKT signaling pathway.<br>Zhu G, Ma B, Dong P, Shang J, Gu X, Zi Y.<br>Cell biology international (2020)<br>PMID 31535749; doi:10.1002/cbin.11240	Controlled animal/mixed preclinical study. Screen-level limitation flag: incomplete reporting of randomization, allocation concealment, blinding, attrition, and prospective sample-size justification.
R0780	2019	Bone loss/osteogenesis<br>Animal	Melatonin promotes the BMP9-induced osteogenic differentiation of mesenchymal stem cells by activating the AMPK/β-catenin signalling pathway.<br>Jiang T, Xia C, Chen X, Hu Y, Wang Y, Wu J, Chen S, Gao Y.<br>Stem cell research & therapy (2019)<br>PMID 31864412; doi:10.1186/s13287-019-1511-7	Controlled animal/mixed preclinical study. Screen-level limitation flag: incomplete reporting of randomization, allocation concealment, blinding, attrition, and prospective sample-size justification.
R0815	2019	Bone loss/osteogenesis<br>Animal	Melatonin prevents bone destruction in mice with retinoic acid-induced osteoporosis.<br>Wang X, Liang T, Zhu Y, Qiu J, Qiu X, Lian C, Gao B, Peng Y, Liang A, Zhou H, Yang X, Liao Z, Li Y, Xu C, Su P, Huang D.<br>Molecular medicine (Cambridge, Mass.) (2019)<br>PMID 31462213; doi:10.1186/s10020-019-0107-0	Controlled animal/mixed preclinical study. Screen-level limitation flag: incomplete reporting of randomization, allocation concealment, blinding, attrition, and prospective sample-size justification.
R0819	2019	Bone loss/osteogenesis<br>Animal	Melatonin prevents cadmium-induced bone damage: First evidence on an improved osteogenic/adipogenic differentiation balance of mesenchymal stem cells as underlying mechanism.<br>Knani L, Bartolini D, Kechiche S, Tortoioli C, Murdolo G, Moretti M, Messaoudi I, Reiter RJ, Galli F.<br>Journal of pineal research (2019)<br>PMID 31340072; doi:10.1111/jpi.12597	Controlled animal/mixed preclinical study. Screen-level limitation flag: incomplete reporting of randomization, allocation concealment, blinding, attrition, and prospective sample-size justification.
R0833	2019	Bone loss/osteogenesis<br>Animal	Suppressive effect of melatonin on osteoclast function via osteocyte calcitonin.<br>Nakano M, Ikegame M, Igarashi-Migitaka J, Maruyama Y, Suzuki N, Hattori A.<br>The Journal of endocrinology (2019)<br>PMID 31042672; doi:10.1530/joe-18-0707	Controlled animal/mixed preclinical study. Screen-level limitation flag: incomplete reporting of randomization, allocation concealment, blinding, attrition, and prospective sample-size justification.
R0900	2018	Bone loss/osteogenesis<br>Animal	Melatonin Suppresses Estrogen Deficiency-Induced Osteoporosis and Promotes Osteoblastogenesis by Inactivating the NLRP3 Inflammasome.<br>Xu L, Zhang L, Wang Z, Li C, Li S, Li L, Fan Q, Zheng L.<br>Calcified tissue international (2018)<br>PMID 29804160; doi:10.1007/s00223-018-0428-y	Controlled animal/mixed preclinical study. Screen-level limitation flag: incomplete reporting of randomization, allocation concealment, blinding, attrition, and prospective sample-size justification.
R0925	2018	Bone loss/osteogenesis<br>Animal	Biological effects of melatonin on osteoblast/osteoclast cocultures, bone, and quality of life: Implications of a role for MT2 melatonin receptors, MEK1/2, and MEK5 in melatonin-mediated osteoblastogenesis.<br>Maria S, Samsonraj RM, Munmun F, Glas J, Silvestros M, Kotlarczyk MP, Rylands R, Dudakovic A, van Wijnen AJ, Enderby LT, Lassila H, Dodda B, Davis VL, Balk J, Burow M, Bunnell BA, Witt-Enderby PA.<br>Journal of pineal research (2018)<br>PMID 29285799; doi:10.1111/jpi.12465	Controlled animal/mixed preclinical study. Screen-level limitation flag: incomplete reporting of randomization, allocation concealment, blinding, attrition, and prospective sample-size justification.

ID	Year	Domain / stream	Report and identifier	Design and screen-level methodological limitation assessment
R0932	2018	Bone loss/osteogenesis<br>Animal	Melatonin therapy reduces the risk of osteoporosis and normalizes bone formation in multiple sclerosis.<br>Ghareghani M, Scavo L, Arnoult D, Zibara K, Farhadi N.<br>Fundamental & clinical pharmacology (2018)<br>PMID 29193274; doi:10.1111/fcp.12337	Controlled animal/mixed preclinical study. Screen-level limitation flag: incomplete reporting of randomization, allocation concealment, blinding, attrition, and prospective sample-size justification.
R0937	2017	Bone loss/osteogenesis<br>Animal	Melatonin promotes osteoblast differentiation of bone marrow mesenchymal stem cells in aged rats.<br>Chu ZM, Li HB, Sun SX, Jiang YC, Wang B, Dong YF.<br>European review for medical and pharmacological sciences (2017)<br>PMID 29077147	Controlled animal/mixed preclinical study. Screen-level limitation flag: incomplete reporting of randomization, allocation concealment, blinding, attrition, and prospective sample-size justification.
R0948	2017	Bone loss/osteogenesis<br>Animal	Inhibitory effects of melatonin on titanium particle-induced inflammatory bone resorption and osteoclastogenesis via suppression of NF-κB signaling.<br>Ping Z, Ping Z, Wang Z, Shi J, Wang L, Guo X, Zhou W, Hu X, Wu X, Liu Y, Zhang W, Yang H, Xu Y, Gu Y, Geng D.<br>Acta biomaterialia (2017)<br>PMID 28867647; doi:10.1016/j.actbio.2017.08.046	Controlled animal/mixed preclinical study. Screen-level limitation flag: incomplete reporting of randomization, allocation concealment, blinding, attrition, and prospective sample-size justification.
R1021	2016	Bone loss/osteogenesis<br>Animal	Melatonin suppresses autophagy in type 2 diabetic osteoporosis.<br>Zhang WL, Meng HZ, Yang RF, Yang MW, Sun GH, Liu JH, Shi PX, Liu F, Yang B.<br>Oncotarget (2016)<br>PMID 27438148; doi:10.18632/oncotarget.10538	Controlled animal/mixed preclinical study. Screen-level limitation flag: incomplete reporting of randomization, allocation concealment, blinding, attrition, and prospective sample-size justification.
R1117	2014	Bone loss/osteogenesis<br>Animal	Melatonin promotes osteoblast differentiation and mineralization of MC3T3-E1 cells under hypoxic conditions through activation of PKD/p38 pathways.<br>Son JH, Cho YC, Sung IY, Kim IR, Park BS, Kim YD.<br>Journal of pineal research (2014)<br>PMID 25250639; doi:10.1111/jpi.12177	Controlled animal/mixed preclinical study. Screen-level limitation flag: incomplete reporting of randomization, allocation concealment, blinding, attrition, and prospective sample-size justification.
R1255	2011	Bone loss/osteogenesis<br>Animal	Melatonin promotes osteoblastic differentiation through the BMP/ERK/Wnt signaling pathways.<br>Park KH, Kang JW, Lee EM, Kim JS, Rhee YH, Kim M, Jeong SJ, Park YG, Kim SH.<br>Journal of pineal research (2011)<br>PMID 21470302; doi:10.1111/j.1600-079x.2011.00875.x	Controlled animal/mixed preclinical study. Screen-level limitation flag: incomplete reporting of randomization, allocation concealment, blinding, attrition, and prospective sample-size justification.
R1273	2010	Bone loss/osteogenesis<br>Animal	Melatonin inhibits adipogenesis and enhances osteogenesis of human mesenchymal stem cells by suppressing PPARγ expression and enhancing Runx2 expression.<br>Zhang L, Su P, Xu C, Chen C, Liang A, Du K, Peng Y, Huang D.<br>Journal of pineal research (2010)<br>PMID 20738756; doi:10.1111/j.1600-079x.2010.00803.x	Controlled animal/mixed preclinical study. Screen-level limitation flag: incomplete reporting of randomization, allocation concealment, blinding, attrition, and prospective sample-size justification.

ID	Year	Domain / stream	Report and identifier	Design and screen-level methodological limitation assessment
R1363	2007	Bone loss/osteogenesis<br>Animal	Constructive effect of exogenous melatonin against osteoporosis after ovariectomy in rats.<br>Uslu S, Uysal A, Oktem G, Yurtseven M, Tanyalçin T, Başdemir G.<br>Analytical and quantitative cytology and histology (2007)<br>PMID 17987812	Controlled animal/mixed preclinical study. Screen-level limitation flag: incomplete reporting of randomization, allocation concealment, blinding, attrition, and prospective sample-size justification.
R1376	2007	Bone loss/osteogenesis<br>Animal	Melatonin inhibits fatty acid-induced triglyceride accumulation in ROS17/2.8 cells: implications for osteoblast differentiation and osteoporosis.<br>Sanchez-Hidalgo M, Lu Z, Tan DX, Maldonado MD, Reiter RJ, Gregerman RI.<br>American journal of physiology. Regulatory, integrative and comparative physiology (2007)<br>PMID 17379847; doi:10.1152/ajpregu.00013.2007	Controlled animal/mixed preclinical study. Screen-level limitation flag: incomplete reporting of randomization, allocation concealment, blinding, attrition, and prospective sample-size justification.
R1474	2002	Bone loss/osteogenesis<br>Animal	The influence of pinealectomy and melatonin administration on the dynamic pattern of biochemical markers of bone metabolism in experimental osteoporosis in the rat.<br>Ostrowska Z, Kos-Kudla B, Marek B, Kajdaniuk D, Staszewicz P, Szapska B, Strzelczyk J.<br>Neuro endocrinology letters (2002)<br>PMID 12019362	Controlled animal/mixed preclinical study. Screen-level limitation flag: incomplete reporting of randomization, allocation concealment, blinding, attrition, and prospective sample-size justification.
R1526	1999	Bone loss/osteogenesis<br>Animal	Melatonin promotes osteoblast differentiation and bone formation.<br>Roth JA, Kim BG, Lin WL, Cho MI.<br>The Journal of biological chemistry (1999)<br>PMID 10419530; doi:10.1074/jbc.274.31.22041	Controlled animal/mixed preclinical study. Screen-level limitation flag: incomplete reporting of randomization, allocation concealment, blinding, attrition, and prospective sample-size justification.
R0031	2026	Bone loss/osteogenesis<br>Human	Melatonin, Caffeine, or Their Combination: Effects on Sleep, Performance, Perceived Exertion in a Placebo-Controlled Crossover Study.<br>Mahdi N, Delleli S, Maaoui KB, Jebabli A, Del Coso J, Chtourou H, Ardigò LP, Ouergui I.<br>Nutrients (2026)<br>PMID 42124027; doi:10.3390/nu18091425	Human randomized crossover. Screen-level limitation flag: small/short trial; incomplete reporting of allocation concealment, multiplicity, or attrition. Outcome is supportive-care or surrogate rather than proof of disease modification.
R0115	2025	Bone loss/osteogenesis<br>Human	Pilot Study Assessing the Efficacy of Melatonin and Osteogenic Loading on Bone Health in a Postmenopausal Population with Osteopenia: MelaOstrong Randomized Controlled Trial.<br>Jahan A, Myoung Y, Malik A, Gionfriddo MR, Witt-Enderby PA.<br>Journal of pineal research (2025)<br>PMID 41059603; doi:10.1111/jpi.70088	Human randomized trial. Screen-level limitation flag: small/short trial; incomplete reporting of allocation concealment, multiplicity, or attrition.
R0183	2025	Bone loss/osteogenesis<br>Human	Melatonin Supplementation Alleviates Bone Mineral Density Decline and Circulating Oxidative Stress in Iron-Overloaded Thalassemia Patients.<br>Piriyakhuntorn P, Tantiworawit A, Phimphilai M, Kaewchur T, Niprapan P, Srivichit B, Apaijai N, Shinlapawittayatorn K, Chattipakorn N, Chattipakorn SC.<br>Journal of pineal research (2025)<br>PMID 40329508; doi:10.1111/jpi.70055	Human randomized trial. Screen-level limitation flag: small/short trial; incomplete reporting of allocation concealment, multiplicity, or attrition. Outcome is supportive-care or surrogate rather than proof of disease modification.

ID	Year	Domain / stream	Report and identifier	Design and screen-level methodological limitation assessment
R1068	2015	Bone loss/osteogenesis<br>Human	The effect of melatonin treatment on postural stability, muscle strength, and quality of life and sleep in postmenopausal women: a randomized controlled trial.<br>Amstrup AK, Sikjaer T, Mosekilde L, Rejnmark L.<br>Nutrition journal (2015)<br>PMID 26424587; doi:10.1186/s12937-015-0093-1	Human randomized trial. Screen-level limitation flag: small/short trial; incomplete reporting of allocation concealment, multiplicity, or attrition. Outcome is supportive-care or surrogate rather than proof of disease modification.
R1082	2015	Bone loss/osteogenesis<br>Human	Melatonin improves bone mineral density at the femoral neck in postmenopausal women with osteopenia: a randomized controlled trial.<br>Amstrup AK, Sikjaer T, Heickendorff L, Mosekilde L, Rejnmark L.<br>Journal of pineal research (2015)<br>PMID 26036434; doi:10.1111/jpi.12252	Human randomized trial. Screen-level limitation flag: small/short trial; incomplete reporting of allocation concealment, multiplicity, or attrition. Outcome is supportive-care or surrogate rather than proof of disease modification.
R1232	2012	Bone loss/osteogenesis<br>Human	Melatonin osteoporosis prevention study (MOPS): a randomized, double-blind, placebo-controlled study examining the effects of melatonin on bone health and quality of life in perimenopausal women.<br>Kotlarczyk MP, Lassila HC, O'Neil CK, D'Amico F, Enderby LT, Witt-Enderby PA, Balk JL.<br>Journal of pineal research (2012)<br>PMID 22220591; doi:10.1111/j.1600-079x.2011.00956.x	Human randomized trial. Screen-level limitation flag: small/short trial; incomplete reporting of allocation concealment, multiplicity, or attrition.
R0100	2026	Bone loss/osteogenesis<br>In vitro	Melatonin promotes osteogenic differentiation of BMSCs by regulating circZFAND1/miR-433-3p/DKK1 pathway through melatonin receptor-dependent m6A modification of circZFAND1.<br>Shi Y, Yu J, Wu Y, Fang H, Yang B.<br>Bone (2026)<br>PMID 41192527; doi:10.1016/j.bone.2025.117691	Controlled in-vitro/cell/tissue study. Screen-level limitation flag: incomplete reporting of concentration justification, independent replication, blinding, and external validity.
R0538	2022	Bone loss/osteogenesis<br>In vitro	Melatonin inhibits osteoclastogenesis via RANKL/OPG suppression mediated by Rev-Erbα in osteoblasts.<br>Tian Y, Ming J.<br>Journal of cellular and molecular medicine (2022)<br>PMID 35726597; doi:10.1111/jcmm.17440	Controlled in-vitro/cell/tissue study. Screen-level limitation flag: incomplete reporting of concentration justification, independent replication, blinding, and external validity.
R0596	2022	Bone loss/osteogenesis<br>In vitro	Melatonin Attenuates RANKL-Induced Osteoclastogenesis via Inhibition of Atp6v0d2 and DC-STAMP through MAPK and NFATc1 Signaling Pathways.<br>Kim SS, Jeong SP, Park BS, Kim IR.<br>Molecules (Basel, Switzerland) (2022)<br>PMID 35056817; doi:10.3390/molecules27020501	Controlled in-vitro/cell/tissue study. Screen-level limitation flag: incomplete reporting of concentration justification, independent replication, blinding, and external validity.
R0672	2021	Bone loss/osteogenesis<br>In vitro	Effect of melatonin/BMP-2 co-delivery scaffolds on the osteoclast activity.<br>Jarrar H, Çetin Altındal D, Gümüşderelioğlu M.<br>Journal of materials science. Materials in medicine (2021)<br>PMID 33751250; doi:10.1007/s10856-021-06502-0	Controlled in-vitro/cell/tissue study. Screen-level limitation flag: incomplete reporting of concentration justification, independent replication, blinding, and external validity.

ID	Year	Domain / stream	Report and identifier	Design and screen-level methodological limitation assessment
R0695	2020	Bone loss/osteogenesis<br>In vitro	The inhibitory effect of melatonin on osteoclastogenesis of RAW 264.7 cells in low concentrations of RANKL and MCSF.<br>Jarrar H, Çetİn Altindal D, GÜmÜŞderelİoĞlu M.<br>Turkish journal of biology = Turk biyoloji dergisi (2020)<br>PMID 33402869; doi:10.3906/biy-2007-85	Controlled in-vitro/cell/tissue study. Screen-level limitation flag: incomplete reporting of concentration justification, independent replication, blinding, and external validity.
R0696	2020	Bone loss/osteogenesis<br>In vitro	Melatonin Inhibits Glucose-Induced Apoptosis in Osteoblastic Cell Line Through PERK-eIF2α-ATF4 Pathway.<br>Zhou R, Ma Y, Tao Z, Qiu S, Gong Z, Tao L, Zhu Y.<br>Frontiers in pharmacology (2020)<br>PMID 33390989; doi:10.3389/fphar.2020.602307	Controlled in-vitro/cell/tissue study. Screen-level limitation flag: incomplete reporting of concentration justification, independent replication, blinding, and external validity.
R0737	2020	Bone loss/osteogenesis<br>In vitro	Melatonin rescues glucocorticoid-induced inhibition of osteoblast differentiation in MC3T3-E1 cells via the PI3K/AKT and BMP/Smad signalling pathways.<br>Zhao R, Tao L, Qiu S, Shen L, Tian Y, Gong Z, Tao ZB, Zhu Y.<br>Life sciences (2020)<br>PMID 32622944; doi:10.1016/j.lfs.2020.118044	Controlled in-vitro/cell/tissue study. Screen-level limitation flag: incomplete reporting of concentration justification, independent replication, blinding, and external validity.
R0765	2020	Bone loss/osteogenesis<br>In vitro	Melatonin induces mitochondrial apoptosis in osteoblasts by regulating the STIM1/cytosolic calcium elevation/ERK pathway.<br>Qiu S, Tao ZB, Tao L, Zhu Y.<br>Life sciences (2020)<br>PMID 32088216; doi:10.1016/j.lfs.2020.117455	Controlled in-vitro/cell/tissue study. Screen-level limitation flag: incomplete reporting of concentration justification, independent replication, blinding, and external validity.
R0810	2020	Bone loss/osteogenesis<br>In vitro	Melatonin Attenuates AlCl3-Induced Apoptosis and Osteoblastic Differentiation Suppression by Inhibiting Oxidative Stress in MC3T3-E1 Cells.<br>Cao Z, Geng X, Jiang X, Gao X, Liu K, Li Y.<br>Biological trace element research (2020)<br>PMID 31502178; doi:10.1007/s12011-019-01893-2	Controlled in-vitro/cell/tissue study. Screen-level limitation flag: incomplete reporting of concentration justification, independent replication, blinding, and external validity.
R0917	2018	Bone loss/osteogenesis<br>In vitro	Melatonin regulates CRE-dependent gene transcription underlying osteoblast proliferation by activating Src and PKA in parallel.<br>Tao L, Zhu Y.<br>American journal of translational research (2018)<br>PMID 29422996	Controlled in-vitro/cell/tissue study. Screen-level limitation flag: incomplete reporting of concentration justification, independent replication, blinding, and external validity.
R0956	2017	Bone loss/osteogenesis<br>In vitro	Melatonin promotes osteoblast differentiation by regulating Osterix protein stability and expression.<br>Han Y, Kim YM, Kim HS, Lee KY.<br>Scientific reports (2017)<br>PMID 28720849; doi:10.1038/s41598-017-06304-x	Controlled in-vitro/cell/tissue study. Screen-level limitation flag: incomplete reporting of concentration justification, independent replication, blinding, and external validity.

ID	Year	Domain / stream	Report and identifier	Design and screen-level methodological limitation assessment
R0970	2017	Bone loss/osteogenesis<br>In vitro	Cytoprotective effects of melatonin on zoledronic acid-treated human osteoblasts.<br>Camacho-Alonso F, Urrutia-Rodríguez I, Oñate-Cabrerizo D, Oñate-Sánchez RE, Rodríguez-Lozano FJ.<br>Journal of cranio-maxillo-facial surgery : official publication of the European Association for Cranio-Maxillo-Facial Surgery (2017)<br>PMID 28552199; doi:10.1016/j.jcms.2017.04.006	Controlled in-vitro/cell/tissue study. Screen-level limitation flag: incomplete reporting of concentration justification, independent replication, blinding, and external validity.
R1085	2015	Bone loss/osteogenesis<br>In vitro	Effect of Melatonin on the Extracellular-Regulated Kinase Signal Pathway Activation and Human Osteoblastic Cell Line hFOB 1.19 Proliferation.<br>Xiong XC, Zhu Y, Ge R, Liu LF, Yuan W.<br>International journal of molecular sciences (2015)<br>PMID 25961946; doi:10.3390/ijms160510337	Controlled in-vitro/cell/tissue study. Screen-level limitation flag: incomplete reporting of concentration justification, independent replication, blinding, and external validity.
R1156	2014	Bone loss/osteogenesis<br>In vitro	Melatonin protects MG63 osteoblast-like cells from hydrogen peroxide-induced cytotoxicity by maintaining mitochondrial function.<br>She F, Wang W, Wang Y, Tang P, Wei J, Chen H, Zhang B.<br>Molecular medicine reports (2014)<br>PMID 24297096; doi:10.3892/mmr.2013.1832	Controlled in-vitro/cell/tissue study. Screen-level limitation flag: incomplete reporting of concentration justification, independent replication, blinding, and external validity.
R1397	2006	Bone loss/osteogenesis<br>In vitro	Melatonin enhances alkaline phosphatase activity in differentiating human adult mesenchymal stem cells grown in osteogenic medium via MT2 melatonin receptors and the MEK/ERK (1/2) signaling cascade.<br>Radio NM, Doctor JS, Witt-Enderby PA.<br>Journal of pineal research (2006)<br>PMID 16635021; doi:10.1111/j.1600-079x.2006.00318.x	Controlled in-vitro/cell/tissue study. Screen-level limitation flag: incomplete reporting of concentration justification, independent replication, blinding, and external validity.
R0233	2024	Exercise/sports recovery<br>Animal	Effects of Melatonin Administration on Physical Performance and Biochemical Responses Following Exhaustive Treadmill Exercise.<br>Berzosa C, Bascuas PJ, Piedrafita E.<br>Current issues in molecular biology (2024)<br>PMID 39727943; doi:10.3390/cimb46120815	Controlled animal/mixed preclinical study. Screen-level limitation flag: incomplete reporting of randomization, allocation concealment, blinding, attrition, and prospective sample-size justification.
R0623	2021	Exercise/sports recovery<br>Animal	Acute melatonin administration improves exercise tolerance and the metabolic recovery after exhaustive effort.<br>Faria VS, Pejon TMM, Gobatto CA, de Araujo GG, Cornachione AS, Beck WR.<br>Scientific reports (2021)<br>PMID 34584111; doi:10.1038/s41598-021-97364-7	Controlled animal/mixed preclinical study. Screen-level limitation flag: incomplete reporting of randomization, allocation concealment, blinding, attrition, and prospective sample-size justification.
R1058	2015	Exercise/sports recovery<br>Animal	Melatonin Has An Ergogenic Effect But Does Not Prevent Inflammation and Damage In Exhaustive Exercise.<br>Beck WR, Botezelli JD, Pauli JR, Ropelle ER, Gobatto CA.<br>Scientific reports (2015)<br>PMID 26669455; doi:10.1038/srep18065	Controlled animal/mixed preclinical study. Screen-level limitation flag: incomplete reporting of randomization, allocation concealment, blinding, attrition, and prospective sample-size justification.

ID	Year	Domain / stream	Report and identifier	Design and screen-level methodological limitation assessment
R1140	2014	Exercise/sports recovery<br>Animal	Salutary effects of melatonin combined with treadmill exercise on cartilage damage.<br>Hong Y, Kim H, Lee Y, Lee S, Kim K, Jin Y, Lee SR, Chang KT, Hong Y.<br>Journal of pineal research (2014)<br>PMID 24816289; doi:10.1111/jpi.12143	Controlled animal/mixed preclinical study. Screen-level limitation flag: incomplete reporting of randomization, allocation concealment, blinding, attrition, and prospective sample-size justification.
R1155	2014	Exercise/sports recovery<br>Animal	Melatonin treatment combined with treadmill exercise accelerates muscular adaptation through early inhibition of CHOP-mediated autophagy in the gastrocnemius of rats with intra-articular collagenase-induced knee laxity.<br>Hong Y, Kim JH, Jin Y, Lee S, Park K, Lee Y, Chang KT, Hong Y.<br>Journal of pineal research (2014)<br>PMID 24313305; doi:10.1111/jpi.12110	Controlled animal/mixed preclinical study. Screen-level limitation flag: incomplete reporting of randomization, allocation concealment, blinding, attrition, and prospective sample-size justification.
R1396	2006	Exercise/sports recovery<br>Animal	Melatonin supplementation to rats subjected to acute swimming exercise: Its effect on plasma lactate levels and relation with zinc.<br>Kaya O, Gokdemir K, Kilic M, Baltaci AK.<br>Neuro endocrinology letters (2006)<br>PMID 16648794	Controlled animal/mixed preclinical study. Screen-level limitation flag: incomplete reporting of randomization, allocation concealment, blinding, attrition, and prospective sample-size justification.
R1484	2001	Exercise/sports recovery<br>Animal	Effects of melatonin on fuel utilization in exercised rats: role of nitric oxide and growth hormone.<br>Sánchez-Campos S, Arévalo M, Mesonero MJ, Esteller A, González-Gallego J, Collado PS.<br>Journal of pineal research (2001)<br>PMID 11555172; doi:10.1034/j.1600-079x.2001.310210.x	Controlled animal/mixed preclinical study. Screen-level limitation flag: incomplete reporting of randomization, allocation concealment, blinding, attrition, and prospective sample-size justification.
R0046	2026	Exercise/sports recovery<br>Human	Improved sleep and heart rate stability with low-dose melatonin in young male athletes following exhaustive exercise.<br>Aykora D, Güngör B, Açak M, Aydeğer C, Özen G, Uzun M.<br>Chronobiology international (2026)<br>PMID 41937599; doi:10.1080/07420528.2026.2650828	Human randomized trial. Screen-level limitation flag: small/short trial; incomplete reporting of allocation concealment, multiplicity, or attrition. Outcome is supportive-care or surrogate rather than proof of disease modification.
R0049	2026	Exercise/sports recovery<br>Human	Acute Effects of Different Melatonin Doses on Performance and Psychophysiological Responses During Exhaustive Cycling Exercise: A Double-Blind Crossover Study.<br>Pedroso LC, Reis MCD, Bertolucci V, Silva LA, Reis IGMD, Beck WR, Scariot PPM, Messias LHD.<br>Nutrients (2026)<br>PMID 41829968; doi:10.3390/nu18050798	Human randomized crossover. Screen-level limitation flag: small/short trial; incomplete reporting of allocation concealment, multiplicity, or attrition.
R0160	2025	Exercise/sports recovery<br>Human	Melatonin Supplementation Enhances Next-Day High-Intensity Exercise Performance and Recovery in Trained Males: A Placebo-Controlled Crossover Study.<br>Mahdi N, Delleli S, Jebabli A, Ben Maaoui K, Del Coso J, Chtourou H, Ardigò LP, Ouergui I.<br>Sports (Basel, Switzerland) (2025)<br>PMID 40559702; doi:10.3390/sports13060190	Human randomized crossover. Screen-level limitation flag: small/short trial; incomplete reporting of allocation concealment, multiplicity, or attrition.

ID	Year	Domain / stream	Report and identifier	Design and screen-level methodological limitation assessment
R0249	2024	Exercise/sports recovery<br>Human	The Synergistic Effect of Intradialytic Concurrent Training and Melatonin Supplementation on Oxidative Stress and Inflammation in Hemodialysis Patients: A Double-Blind Randomized Controlled Trial.<br>Marzougui H, Ben Dhia I, Mezghani I, Maaloul R, Toumi S, Kammoun K, Chaabouni MN, Ayadi F, Ben Hmida M, Turki M, Hammouda O.<br>Antioxidants (Basel, Switzerland) (2024)<br>PMID 39594432; doi:10.3390/antiox13111290	Human randomized trial. Screen-level limitation flag: small/short trial; incomplete reporting of allocation concealment, multiplicity, or attrition. Outcome is supportive-care or surrogate rather than proof of disease modification.
R0276	2024	Exercise/sports recovery<br>Human	Effects of Melatonin on Exercise-Induced Oxidative Stress in Adults with Obesity Undergoing a Multidisciplinary Body Weight Reduction Program.<br>Rigamonti AE, Rubino FM, Caroli D, Bondesan A, Mai S, Cella SG, Centofanti L, Paroni R, Sartorio A.<br>Journal of clinical medicine (2024)<br>PMID 39274429; doi:10.3390/jcm13175216	Human randomized trial. Screen-level limitation flag: small/short trial; incomplete reporting of allocation concealment, multiplicity, or attrition. Outcome is supportive-care or surrogate rather than proof of disease modification.
R0473	2023	Exercise/sports recovery<br>Human	Effects of intradialytic exercise in combination with melatonin supplementation on functional capacity, postural balance, and quality of life in hemodialysis patients.<br>Marzougui H, Maaloul R, Ben Dhia I, Toumi S, Kammoun K, Ben Hmida M, Ayadi F, Turki M, Elleuch MH, Ghroubi S, Hammouda O.<br>Therapeutic apheresis and dialysis : official peer-reviewed journal of the International Society for Apheresis, the Japanese Society for Apheresis, the Japanese Society for Dialysis Therapy (2023)<br>PMID 36579842; doi:10.1111/1744-9987.13964	Human randomized trial. Screen-level limitation flag: small/short trial; incomplete reporting of allocation concealment, multiplicity, or attrition.
R0569	2022	Exercise/sports recovery<br>Human	Effects of melatonin ingestion on physical performance and biochemical responses following exhaustive running exercise in soccer players.<br>Farjallah MA, Graja A, Mahmoud LB, Ghattassi K, Boudaya M, Driss T, Jamoussi K, Sahnoun Z, Souissi N, Hammouda O.<br>Biology of sport (2022)<br>PMID 35309526; doi:10.5114/biolsport.2022.106385	Human randomized trial. Screen-level limitation flag: small/short trial; incomplete reporting of allocation concealment, multiplicity, or attrition.
R0663	2021	Exercise/sports recovery<br>Human	Melatonin supplementation improves psychomotor and physical performance in collegiate student-athletes following a sleep deprivation night.<br>Paryab N, Taheri M, H'Mida C, Irandoust K, Mirmoezzi M, Trabelsi K, Ammar A, Chtourou H.<br>Chronobiology international (2021)<br>PMID 33845710; doi:10.1080/07420528.2021.1889578	Human randomized trial. Screen-level limitation flag: small/short trial; incomplete reporting of allocation concealment, multiplicity, or attrition. Outcome is supportive-care or surrogate rather than proof of disease modification.
R0752	2020	Exercise/sports recovery<br>Human	Effect of nocturnal melatonin intake on cellular damage and recovery from repeated sprint performance during an intensive training schedule.<br>Farjallah MA, Ghattassi K, Ben Mahmoud L, Graja A, Boudaya M, Elleuch H, Jammoussi K, Sahnoun Z, Souissi N, Chtourou H, Hammouda O.<br>Chronobiology international (2020)<br>PMID 32378422; doi:10.1080/07420528.2020.1746797	Human randomized trial. Screen-level limitation flag: small/short trial; incomplete reporting of allocation concealment, multiplicity, or attrition.
R0855	2019	Exercise/sports recovery<br>Human	Melatonin supplementation improves oxidative and inflammatory state in the blood of professional athletes during the preparatory period for competitions.<br>Czuczejko J, Sielski Ł, Woźniak B, Woźniak A, Szewczyk-Golec K.<br>Free radical research (2019)<br>PMID 30646765; doi:10.1080/10715762.2018.1563688	Human comparative study. Screen-level limitation flag: incomplete reporting of comparative design and prespecified analysis. Outcome is supportive-care or surrogate rather than proof of disease modification.

ID	Year	Domain / stream	Report and identifier	Design and screen-level methodological limitation assessment
R0859	2018	Exercise/sports recovery<br>Human	Effect of melatonin ingestion on physical performance, metabolic responses, and recovery after an intermittent training session.<br>Farjallah MA, Hammouda O, Zouch M, Ghattassi K, Graja A, Driss T, Chamari K, Souissi N.<br>Physiology international (2018)<br>PMID 30560679; doi:10.1556/2060.105.2018.4.24	Human randomized trial. Screen-level limitation flag: small/short trial; incomplete reporting of allocation concealment, multiplicity, or attrition.
R0899	2018	Exercise/sports recovery<br>Human	Melatonin ingestion after exhaustive late-evening exercise improves sleep quality and quantity, and short-term performances in teenage athletes.<br>Cheikh M, Hammouda O, Gaamouri N, Driss T, Chamari K, Cheikh RB, Dogui M, Souissi N.<br>Chronobiology international (2018)<br>PMID 29846091; doi:10.1080/07420528.2018.1474891	Human randomized trial. Screen-level limitation flag: small/short trial; incomplete reporting of allocation concealment, multiplicity, or attrition. Outcome is supportive-care or surrogate rather than proof of disease modification.
R0969	2018	Exercise/sports recovery<br>Human	Consumption of a 5-mg Melatonin Supplement Does Not Affect 32.2-km Cycling Time Trial Performance.<br>Brandenberger KJ, Ingalls CP, Rupp JC, Doyle JA.<br>Journal of strength and conditioning research (2018)<br>PMID 28557857; doi:10.1519/jsc.0000000000001955	Human randomized crossover. Screen-level limitation flag: small/short trial; incomplete reporting of allocation concealment, multiplicity, or attrition.
R0934	2017	Exercise/sports recovery<br>Human	Effect of Melatonin Supplementation on Antioxidant Status and DNA Damage in High Intensity Trained Athletes.<br>Ortiz-Franco M, Planells E, Quintero B, Acuña-Castroviejo D, Rusanova I, Escames G, Molina-López J.<br>International journal of sports medicine (2017)<br>PMID 29165732; doi:10.1055/s-0043-119881	Human randomized trial. Screen-level limitation flag: small/short trial; incomplete reporting of allocation concealment, multiplicity, or attrition.
R0985	2017	Exercise/sports recovery<br>Human	The benefit of a supplement with the antioxidant melatonin on redox status and muscle damage in resistance-trained athletes.<br>Leonardo-Mendonça RC, Ocaña-Wilhelmi J, de Haro T, de Teresa-Galván C, Guerra-Hernández E, Rusanova I, Fernández-Ortiz M, Sayed RKA, Escames G, Acuña-Castroviejo D.<br>Applied physiology, nutrition, and metabolism = Physiologie appliquee, nutrition et metabolisme (2017)<br>PMID 28192673; doi:10.1139/apnm-2016-0677	Human randomized trial. Screen-level limitation flag: small/short trial; incomplete reporting of allocation concealment, multiplicity, or attrition.
R1070	2015	Exercise/sports recovery<br>Human	The benefits of four weeks of melatonin treatment on circadian patterns in resistance-trained athletes.<br>Leonardo-Mendonça RC, Martinez-Nicolas A, de Teresa Galván C, Ocaña-Wilhelmi J, Rusanova I, Guerra-Hernández E, Escames G, Acuña-Castroviejo D.<br>Chronobiology international (2015)<br>PMID 26361788; doi:10.3109/07420528.2015.1069830	Human randomized trial. Screen-level limitation flag: small/short trial; incomplete reporting of allocation concealment, multiplicity, or attrition.
R1234	2012	Exercise/sports recovery<br>Human	Melatonin administrated immediately before an intense exercise reverses oxidative stress, improves immunological defenses and lipid metabolism in football players.<br>Maldonado MD, Manfredi M, Ribas-Serna J, Garcia-Moreno H, Calvo JR.<br>Physiology & behavior (2012)<br>PMID 22212240; doi:10.1016/j.physbeh.2011.12.015	Human randomized trial. Screen-level limitation flag: small/short trial; incomplete reporting of allocation concealment, multiplicity, or attrition. Outcome is supportive-care or surrogate rather than proof of disease modification.

ID	Year	Domain / stream	Report and identifier	Design and screen-level methodological limitation assessment
R1252	2011	Exercise/sports recovery<br>Human	Melatonin supplementation ameliorates oxidative stress and inflammatory signaling induced by strenuous exercise in adult human males.<br>Ochoa JJ, Díaz-Castro J, Kajarabille N, García C, Guisado IM, De Teresa C, Guisado R.<br>Journal of pineal research (2011)<br>PMID 21615492; doi:10.1111/j.1600-079x.2011.00899.x	Human randomized trial. Screen-level limitation flag: small/short trial; incomplete reporting of allocation concealment, multiplicity, or attrition. Outcome is supportive-care or surrogate rather than proof of disease modification.
R1364	2007	Exercise/sports recovery<br>Human	Effects of a single dose of N-Acetyl-5-methoxytryptamine (Melatonin) and resistance exercise on the growth hormone/IGF-1 axis in young males and females.<br>Nassar E, Mulligan C, Taylor L, Kerksick C, Galbreath M, Greenwood M, Kreider R, Willoughby DS.<br>Journal of the International Society of Sports Nutrition (2007)<br>PMID 17956623; doi:10.1186/1550-2783-4-14	Human randomized trial. Screen-level limitation flag: small/short trial; incomplete reporting of allocation concealment, multiplicity, or attrition.
R1478	2002	Exercise/sports recovery<br>Human	A multifactorial approach employing melatonin to accelerate resynchronization of sleep-wake cycle after a 12 time-zone westerly transmeridian flight in elite soccer athletes.<br>Cardinali DP, Bortman GP, Liotta G, Pérez Lloret S, Albornoz LE, Cutrera RA, Batista J, Ortega Gallo P.<br>Journal of pineal research (2002)<br>PMID 11841599; doi:10.1034/j.1600-079x.2002.10820.x	Human comparative study. Screen-level limitation flag: incomplete reporting of comparative design and prespecified analysis. Outcome is supportive-care or surrogate rather than proof of disease modification.
R1491	2001	Exercise/sports recovery<br>Human	Are there hangover-effects on physical performance when melatonin is ingested by athletes before nocturnal sleep?<br>Atkinson G, Buckley P, Edwards B, Reilly T, Waterhouse J.<br>International journal of sports medicine (2001)<br>PMID 11354528; doi:10.1055/s-2001-16387	Human randomized trial. Screen-level limitation flag: small/short trial; incomplete reporting of allocation concealment, multiplicity, or attrition. Outcome is supportive-care or surrogate rather than proof of disease modification.
R0077	2026	Fracture/bone repair<br>Animal	Integrating BMSC-inlaid GelMA hydrogels and melatonin-loaded PLGA microspheres into silk fibroin/nanohydroxyapatite scaffolds to repair osteoporotic bone defects.<br>Lv J, Ma B, Li J, Wang Z.<br>Journal of orthopaedic surgery and research (2026)<br>PMID 41491499; doi:10.1186/s13018-025-06590-6	Controlled animal/mixed preclinical study. Screen-level limitation flag: incomplete reporting of randomization, allocation concealment, blinding, attrition, and prospective sample-size justification.
R0068	2025	Fracture/bone repair<br>Animal	Histopathological comparison of the effect of 5% melatonin gel and 1.2% rosuvastatin gel on bone regeneration in the rat model.<br>Hagh LG, Rahimi N, Rezaie A, Yousefimanesh H, Samie N.<br>Dental research journal (2025)<br>PMID 41607737; doi:10.4103/drj.drj_680_23	Controlled animal/mixed preclinical study. Screen-level limitation flag: incomplete reporting of randomization, allocation concealment, blinding, attrition, and prospective sample-size justification.
R0220	2025	Fracture/bone repair<br>Animal	The Effect of Local Melatonin Application on Bone Fracture Healing in Rat Tibias.<br>Tanrisever M, Tekin B, Can UK, Istek O, Ozcan EC, Ozercan IH, Gelic T, Dundar S.<br>Medicina (Kaunas, Lithuania) (2025)<br>PMID 39859128; doi:10.3390/medicina61010146	Controlled animal/mixed preclinical study. Screen-level limitation flag: incomplete reporting of randomization, allocation concealment, blinding, attrition, and prospective sample-size justification.

ID	Year	Domain / stream	Report and identifier	Design and screen-level methodological limitation assessment
R0283	2024	Fracture/bone repair<br>Animal	Melatonin-loaded bioactive microspheres accelerate aged bone regeneration by formation of tunneling nanotubes to enhance mitochondrial transfer.<br>Xiong H, Qiu H, Wang C, Qiu Y, Tan S, Chen K, Zhao F, Song J.<br>Materials today. Bio (2024)<br>PMID 39171100; doi:10.1016/j.mtbio.2024.101175	Controlled animal/mixed preclinical study. Screen-level limitation flag: incomplete reporting of randomization, allocation concealment, blinding, attrition, and prospective sample-size justification.
R0315	2024	Fracture/bone repair<br>Animal	A sponge-like nanofiber melatonin-loaded scaffold accelerates vascularized bone regeneration via improving mitochondrial energy metabolism.<br>Lv N, Hou M, Deng L, Hua X, Zhou X, Liu H, Zhu X, Xu Y, Qian Z, Li Q, Liu M, He F.<br>Materials today. Bio (2024)<br>PMID 38765244; doi:10.1016/j.mtbio.2024.101078	Controlled animal/mixed preclinical study. Screen-level limitation flag: incomplete reporting of randomization, allocation concealment, blinding, attrition, and prospective sample-size justification.
R0376	2024	Fracture/bone repair<br>Animal	Melatonin rescues the mitochondrial function of bone marrow-derived mesenchymal stem cells and improves the repair of osteoporotic bone defect in ovariectomized rats.<br>Gu C, Zhou Q, Hu X, Ge X, Hou M, Wang W, Liu H, Shi Q, Xu Y, Zhu X, Yang H, Chen X, Liu T, He F.<br>Journal of pineal research (2024)<br>PMID 37941528; doi:10.1111/jpi.12924	Controlled animal/mixed preclinical study. Screen-level limitation flag: incomplete reporting of randomization, allocation concealment, blinding, attrition, and prospective sample-size justification.
R0407	2023	Fracture/bone repair<br>Animal	Histopathological evaluation of the effect of melatonin gel on bone regeneration in rat model.<br>Golpasandhagh L, Samie N, Sabiza S, Rezaie A.<br>Journal of Indian Society of Periodontology (2023)<br>PMID 37593555; doi:10.4103/jisp.jisp_169_22	Controlled animal/mixed preclinical study. Screen-level limitation flag: incomplete reporting of randomization, allocation concealment, blinding, attrition, and prospective sample-size justification.
R0479	2023	Fracture/bone repair<br>Animal	Melatonin promotes the restoration of bone defects via enhancement of miR-335-5p combined with inhibition of TNFα/NF-κB signaling.<br>Li Y, Guan H, Tian R, Kong N, Liu G, Li Z, Wang K, Yang P.<br>FASEB journal : official publication of the Federation of American Societies for Experimental Biology (2023)<br>PMID 36520091; doi:10.1096/fj.202200572rrr	Controlled animal/mixed preclinical study. Screen-level limitation flag: incomplete reporting of randomization, allocation concealment, blinding, attrition, and prospective sample-size justification.
R0875	2018	Fracture/bone repair<br>Animal	Melatonin Induces Osteoblastic Differentiation of Mesenchymal Stem Cells and Promotes Fracture Healing in a Rat Model of Femoral Fracture via Neuropeptide Y/Neuropeptide Y Receptor Y1 Signaling.<br>Dong P, Gu X, Zhu G, Li M, Ma B, Zi Y.<br>Pharmacology (2018)<br>PMID 30227410; doi:10.1159/000492576	Controlled animal/mixed preclinical study. Screen-level limitation flag: incomplete reporting of randomization, allocation concealment, blinding, attrition, and prospective sample-size justification.
R1023	2016	Fracture/bone repair<br>Animal	The effects of supplemental melatonin administration on the healing of bone defects in streptozotocin-induced diabetic rats.<br>Yildirimturk S, Batu S, Alatli C, Olgac V, Firat D, Sirin Y.<br>Journal of applied oral science : revista FOB (2016)<br>PMID 27383705; doi:10.1590/1678-775720150570	Controlled animal/mixed preclinical study. Screen-level limitation flag: incomplete reporting of randomization, allocation concealment, blinding, attrition, and prospective sample-size justification.

ID	Year	Domain / stream	Report and identifier	Design and screen-level methodological limitation assessment
R1137	2014	Fracture/bone repair<br>Animal	The effects of melatonin and caffeic acid phenethyl ester (CAPE) on fracture healing under ischemic conditions.<br>Erdem M, Gülabi D, Aşçı M, Bostan B, Güneş T, Köseoğlu RD.<br>Acta orthopaedica et traumatologica turcica (2014)<br>PMID 24901927; doi:10.3944/aott.2014.3244	Controlled animal/mixed preclinical study. Screen-level limitation flag: incomplete reporting of randomization, allocation concealment, blinding, attrition, and prospective sample-size justification.
R1229	2013	Fracture/bone repair<br>Animal	Melatonin promotes angiogenesis during repair of bone defects: a radiological and histomorphometric study in rabbit tibiae.<br>Ramírez-Fernández MP, Calvo-Guirado JL, de-Val JE, Delgado-Ruiz RA, Negri B, Pardo-Zamora G, Peñarrocha D, Barona C, Granero JM, Alcaraz-Baños M.<br>Clinical oral investigations (2013)<br>PMID 22323056; doi:10.1007/s00784-012-0684-6	Controlled animal/mixed preclinical study. Screen-level limitation flag: incomplete reporting of randomization, allocation concealment, blinding, attrition, and prospective sample-size justification.
R1272	2012	Fracture/bone repair<br>Animal	Melatonin impairs fracture healing by suppressing RANKL-mediated bone remodeling.<br>Histing T, Anton C, Scheuer C, Garcia P, Holstein JH, Klein M, Matthys R, Pohlemann T, Menger MD.<br>The Journal of surgical research (2012)<br>PMID 20888595; doi:10.1016/j.jss.2010.08.036	Controlled animal/mixed preclinical study. Screen-level limitation flag: incomplete reporting of randomization, allocation concealment, blinding, attrition, and prospective sample-size justification. Direction conflicts with generic healing claims.
R1268	2010	Fracture/bone repair<br>Animal	Melatonin promotes fracture healing in the rat model.<br>Halıcı M, Öner M, Güney A, Canöz Ö, Narin F, Halıcı C.<br>Eklem hastaliklari ve cerrahisi = Joint diseases & related surgery (2010)<br>PMID 21067500	Controlled animal/mixed preclinical study. Screen-level limitation flag: incomplete reporting of randomization, allocation concealment, blinding, attrition, and prospective sample-size justification.
R0445	2023	Fracture/bone repair<br>Human	The association of melatonin use and hip fracture: a matched cohort study.<br>Yang T, Wu J, Ding X, Zhou B, Xiong Y.<br>Osteoporosis international : a journal established as result of cooperation between the European Foundation for Osteoporosis and the National Osteoporosis Foundation of the USA (2023)<br>PMID 37036474; doi:10.1007/s00198-023-06740-8	Human cohort. Screen-level limitation flag: nonrandomized exposure with potential confounding; outcomes may be indirect.
R0054	2026	Intervertebral-disc degeneration<br>Animal	Melatonin attenuates oxidative stress-induced ferroptosis of nucleus pulposus cells and intervertebral disc degeneration via PI3K/AKT-mTOR pathway.<br>Deng Z, Wang L, Yu T, Liang G, Wang Z, Zhao X, Zhang Z, Long X, Cheng X, Lyu FJ, Yu P, Ning C, Chang Y, Huang Y, Chen C.<br>International journal of surgery (London, England) (2026)<br>PMID 41729690; doi:10.1097/js9.0000000000004980	Controlled animal/mixed preclinical study. Screen-level limitation flag: incomplete reporting of randomization, allocation concealment, blinding, attrition, and prospective sample-size justification.
R0132	2025	Intervertebral-disc degeneration<br>Animal	Melatonin attenuates intervertebral disc degeneration by restoring mitochondrial homeostasis through PGC-1α signaling pathway.<br>Lan T, Yang W, Yan B, Guo W, Zhang Y.<br>Cellular and molecular life sciences : CMLS (2025)<br>PMID 40886189; doi:10.1007/s00018-025-05877-5	Controlled animal/mixed preclinical study. Screen-level limitation flag: incomplete reporting of randomization, allocation concealment, blinding, attrition, and prospective sample-size justification.

ID	Year	Domain / stream	Report and identifier	Design and screen-level methodological limitation assessment
R0236	2024	Intervertebral-disc degeneration<br>Animal	Melatonin mitigates intervertebral disc degeneration by suppressing NLRP3 inflammasome activation via the EGR1/DDX3X pathway.<br>Zhao K, Zhang Y, Liao Z, Zhang W, Li G, Shi P, Cheng Z, Chen Y, Li S, Wang K, Song Y, Feng X, An R, Yang C.<br>FASEB journal : official publication of the Federation of American Societies for Experimental Biology (2024)<br>PMID 39708233; doi:10.1096/fj.202302453rrr	Controlled animal/mixed preclinical study. Screen-level limitation flag: incomplete reporting of randomization, allocation concealment, blinding, attrition, and prospective sample-size justification.
R0254	2024	Intervertebral-disc degeneration<br>Animal	Melatonin attenuates degenerative disc degression by downregulating DLX5 via the TGF/Smad2/3 pathway in nucleus pulposus cells.<br>Zhang K, Wang H, Mo L, Huang X, Yuan C, Liu C.<br>JOR spine (2024)<br>PMID 39539538; doi:10.1002/jsp2.70014	Controlled animal/mixed preclinical study. Screen-level limitation flag: incomplete reporting of randomization, allocation concealment, blinding, attrition, and prospective sample-size justification.
R0385	2023	Intervertebral-disc degeneration<br>Animal	Melatonin-loaded self-healing hydrogel targets mitochondrial energy metabolism and promotes annulus fibrosus regeneration.<br>Hu X, Tian X, Yang C, Ling F, Liu H, Zhu X, Pei M, Yang H, Liu T, Xu Y, He F.<br>Materials today. Bio (2023)<br>PMID 37810753; doi:10.1016/j.mtbio.2023.100811	Controlled animal/mixed preclinical study. Screen-level limitation flag: incomplete reporting of randomization, allocation concealment, blinding, attrition, and prospective sample-size justification.
R0748	2020	Intervertebral-disc degeneration<br>Animal	Melatonin activates autophagy via the NF-κB signaling pathway to prevent extracellular matrix degeneration in intervertebral disc.<br>Chen F, Liu H, Wang X, Li Z, Zhang J, Pei Y, Zheng Z, Wang J.<br>Osteoarthritis and cartilage (2020)<br>PMID 32470597; doi:10.1016/j.joca.2020.05.011	Controlled animal/mixed preclinical study. Screen-level limitation flag: incomplete reporting of randomization, allocation concealment, blinding, attrition, and prospective sample-size justification.
R0778	2019	Intervertebral-disc degeneration<br>Animal	Melatonin Protects Intervertebral Disc from Degeneration by Improving Cell Survival and Function via Activation of the ERK1/2 Signaling Pathway.<br>Ge J, Zhou Q, Niu J, Wang Y, Yan Q, Wu C, Qian J, Yang H, Zou J.<br>Oxidative medicine and cellular longevity (2019)<br>PMID 31885798; doi:10.1155/2019/5120275	Controlled animal/mixed preclinical study. Screen-level limitation flag: incomplete reporting of randomization, allocation concealment, blinding, attrition, and prospective sample-size justification.
R0790	2019	Intervertebral-disc degeneration<br>Animal	Melatonin modulates IL-1β-induced extracellular matrix remodeling in human nucleus pulposus cells and attenuates rat intervertebral disc degeneration and inflammation.<br>Zhang Y, He F, Chen Z, Su Q, Yan M, Zhang Q, Tan J, Qian L, Han Y.<br>Aging (2019)<br>PMID 31772145; doi:10.18632/aging.102472	Controlled animal/mixed preclinical study. Screen-level limitation flag: incomplete reporting of randomization, allocation concealment, blinding, attrition, and prospective sample-size justification.
R0856	2019	Intervertebral-disc degeneration<br>Animal	Melatonin ameliorates intervertebral disc degeneration via the potential mechanisms of mitophagy induction and apoptosis inhibition.<br>Chen Y, Wu Y, Shi H, Wang J, Zheng Z, Chen J, Chen X, Zhang Z, Xu D, Wang X, Xiao J.<br>Journal of cellular and molecular medicine (2019)<br>PMID 30609271; doi:10.1111/jcmm.14125	Controlled animal/mixed preclinical study. Screen-level limitation flag: incomplete reporting of randomization, allocation concealment, blinding, attrition, and prospective sample-size justification.

ID	Year	Domain / stream	Report and identifier	Design and screen-level methodological limitation assessment
R1404	2006	Intervertebral-disc degeneration<br>Animal	The effect of exogenous melatonin administration on trabecular width, ligament thickness and TGF-beta(1) expression in degenerated intervertebral disk tissue in the rat.<br>Turgut M, Oktem G, Uslu S, Yurtseven ME, Aktuğ H, Uysal A.<br>Journal of clinical neuroscience : official journal of the Neurosurgical Society of Australasia (2006)<br>PMID 16546388; doi:10.1016/j.jocn.2005.03.037	Controlled animal/mixed preclinical study. Screen-level limitation flag: incomplete reporting of randomization, allocation concealment, blinding, attrition, and prospective sample-size justification.
R1407	2006	Intervertebral-disc degeneration<br>Animal	Evaluation of the relationship between inducible nitric oxide synthase (iNOS) activity and effects of melatonin in experimental osteoporosis in the rat.<br>Oktem G, Uslu S, Vatansever SH, Aktug H, Yurtseven ME, Uysal A.<br>Surgical and radiologic anatomy : SRA (2006)<br>PMID 16362227; doi:10.1007/s00276-005-0065-9	Controlled animal/mixed preclinical study. Screen-level limitation flag: incomplete reporting of randomization, allocation concealment, blinding, attrition, and prospective sample-size justification.
R1449	2003	Intervertebral-disc degeneration<br>Animal	Changes in vascularity of cartilage endplate of degenerated intervertebral discs in response to melatonin administration in rats.<br>Turgut M, Uslu S, Uysal A, Yurtseven ME, Ustün H.<br>Neurosurgical review (2003)<br>PMID 12962300; doi:10.1007/s10143-003-0259-8	Controlled animal/mixed preclinical study. Screen-level limitation flag: incomplete reporting of randomization, allocation concealment, blinding, attrition, and prospective sample-size justification.
R0005	2026	Intervertebral-disc degeneration<br>In vitro	Melatonin improves mitochondrial function and redox balance in human cartilage endplate-derived stem cells.<br>Mo P, Chen R, Sun L, Chen Y, Xiong J, Wu Y, Xu C.<br>Cytotechnology (2026)<br>PMID 42459259; doi:10.1007/s10616-026-01025-4	Controlled in-vitro/cell/tissue study. Screen-level limitation flag: incomplete reporting of concentration justification, independent replication, blinding, and external validity.
R0618	2021	Intervertebral-disc degeneration<br>In vitro	Melatonin Suppresses Apoptosis of Nucleus Pulposus Cells through Inhibiting Autophagy via the PI3K/Akt Pathway in a High-Glucose Culture.<br>Li J, Wang C, Xue L, Zhang F, Liu J.<br>BioMed research international (2021)<br>PMID 34660789; doi:10.1155/2021/4604258	Controlled in-vitro/cell/tissue study. Screen-level limitation flag: incomplete reporting of concentration justification, independent replication, blinding, and external validity.
R0711	2020	Intervertebral-disc degeneration<br>In vitro	Melatonin prevents the binding of vascular endothelial growth factor to its receptor and promotes the expression of extracellular matrix-associated genes in nucleus pulposus cells.<br>Shen C, Li Y, Chen Y, Huang L, Zhang F, Wu W.<br>Experimental and therapeutic medicine (2020)<br>PMID 32989385; doi:10.3892/etm.2020.9227	Controlled in-vitro/cell/tissue study. Screen-level limitation flag: incomplete reporting of concentration justification, independent replication, blinding, and external validity.
R0061	2026	Osteoarthritis/cartilage<br>Animal	Melatonin Improves Diabetic-Accelerated Experimental Osteoarthritis by Inhibiting NLRP3-Mediated Chondrocyte Pyroptosis via PKG/Nrf2.<br>Yang W, Chen H, Zhang Y, Wang S, Miao X, Qiu Z, Qiu D, Gu B, Zhang L, Zhang J, Ma T.<br>Journal of pineal research (2026)<br>PMID 41704066; doi:10.1111/jpi.70113	Controlled animal/mixed preclinical study. Screen-level limitation flag: incomplete reporting of randomization, allocation concealment, blinding, attrition, and prospective sample-size justification.

ID	Year	Domain / stream	Report and identifier	Design and screen-level methodological limitation assessment
R0071	2026	Osteoarthritis/cartilage<br>Animal	Melatonin Attenuates Synovial Hyperplasia, Inflammation, and Fibrosis and Postpones Osteoarthritis Progression.<br>Guan H, Cao R, Zhang J, Liu C, Duan X, Zhao Y, Xing F, Kong N, Li Y, Wu Z, Li H, Chen Y, Wang K, Tian R, Yang P.<br>FASEB journal : official publication of the Federation of American Societies for Experimental Biology (2026)<br>PMID 41562301; doi:10.1096/fj.202502106r	Controlled animal/mixed preclinical study. Screen-level limitation flag: incomplete reporting of randomization, allocation concealment, blinding, attrition, and prospective sample-size justification.
R0102	2026	Osteoarthritis/cartilage<br>Animal	Melatonin Exerts Chondroprotective Effects Against Osteoarthritis by Promoting PI3K/AKT/FoxO3-Mediated Mitophagy.<br>Huang C, Zhang G, Ma YK, Ma XN, Lv SC.<br>The Kaohsiung journal of medical sciences (2026)<br>PMID 41190713; doi:10.1002/kjm2.70131	Controlled animal/mixed preclinical study. Screen-level limitation flag: incomplete reporting of randomization, allocation concealment, blinding, attrition, and prospective sample-size justification.
R0433	2023	Osteoarthritis/cartilage<br>Animal	Preparation of Melatonin-Loaded Nanoparticles with Targeting and Sustained Release Function and Their Application in Osteoarthritis.<br>Liang H, Yan Y, Sun W, Ma X, Su Z, Liu Z, Chen Y, Yu B.<br>International journal of molecular sciences (2023)<br>PMID 37240086; doi:10.3390/ijms24108740	Controlled animal/mixed preclinical study. Screen-level limitation flag: incomplete reporting of randomization, allocation concealment, blinding, attrition, and prospective sample-size justification.
R0483	2023	Osteoarthritis/cartilage<br>Animal	Melatonin ameliorates osteoarthritis rat cartilage injury by inhibiting matrix metalloproteinases and JAK2/STAT3 signaling pathway.<br>Zhao Z, Bi B, Cheng G, Zhao Y, Wu H, Zheng M, Cao Z.<br>Inflammopharmacology (2023)<br>PMID 36427113; doi:10.1007/s10787-022-01102-y	Controlled animal/mixed preclinical study. Screen-level limitation flag: incomplete reporting of randomization, allocation concealment, blinding, attrition, and prospective sample-size justification.
R0501	2022	Osteoarthritis/cartilage<br>Animal	Melatonin Prevents Chondrocyte Matrix Degradation in Rats with Experimentally Induced Osteoarthritis by Inhibiting Nuclear Factor-κB via SIRT1.<br>Zhao M, Song X, Chen H, Ma T, Tang J, Wang X, Yu Y, Lv L, Jia L, Gao L.<br>Nutrients (2022)<br>PMID 36235621; doi:10.3390/nu14193966	Controlled animal/mixed preclinical study. Screen-level limitation flag: incomplete reporting of randomization, allocation concealment, blinding, attrition, and prospective sample-size justification.
R0519	2022	Osteoarthritis/cartilage<br>Animal	Melatonin promotes sirtuin 1 expression and inhibits IRE1α-XBP1S-CHOP to reduce endoplasmic reticulum stress-mediated apoptosis in chondrocytes.<br>Qin K, Tang H, Ren Y, Yang D, Li Y, Huang W, Wu Y, Yin Z.<br>Frontiers in pharmacology (2022)<br>PMID 36034777; doi:10.3389/fphar.2022.940629	Controlled animal/mixed preclinical study. Screen-level limitation flag: incomplete reporting of randomization, allocation concealment, blinding, attrition, and prospective sample-size justification.
R0525	2022	Osteoarthritis/cartilage<br>Animal	Melatonin Attenuates the Progression of Osteoarthritis in Rats by Inhibiting Inflammation and Related Oxidative Stress on the Surface of Knee Cartilage.<br>Ke C, Li H, Yang D, Ying H, Zhu H, Wang J, Xu J, Wang L.<br>Orthopaedic surgery (2022)<br>PMID 35894841; doi:10.1111/os.13408	Controlled animal/mixed preclinical study. Screen-level limitation flag: incomplete reporting of randomization, allocation concealment, blinding, attrition, and prospective sample-size justification.

ID	Year	Domain / stream	Report and identifier	Design and screen-level methodological limitation assessment
R0540	2022	Osteoarthritis/cartilage<br>Animal	Recharge of chondrocyte mitochondria by sustained release of melatonin protects cartilage matrix homeostasis in osteoarthritis.<br>Zhang Y, Hou M, Liu Y, Liu T, Chen X, Shi Q, Geng D, Yang H, He F, Zhu X.<br>Journal of pineal research (2022)<br>PMID 35726138; doi:10.1111/jpi.12815	Controlled animal/mixed preclinical study. Screen-level limitation flag: incomplete reporting of randomization, allocation concealment, blinding, attrition, and prospective sample-size justification.
R0583	2022	Osteoarthritis/cartilage<br>Animal	Melatonin Prevents Cartilage Degradation in Early-Stage Osteoarthritis Through Activation of miR-146a/NRF2/HO-1 Axis.<br>Zhou X, Zhang Y, Hou M, Liu H, Yang H, Chen X, Liu T, He F, Zhu X.<br>Journal of bone and mineral research : the official journal of the American Society for Bone and Mineral Research (2022)<br>PMID 35147250; doi:10.1002/jbmr.4527	Controlled animal/mixed preclinical study. Screen-level limitation flag: incomplete reporting of randomization, allocation concealment, blinding, attrition, and prospective sample-size justification.
R0613	2021	Osteoarthritis/cartilage<br>Animal	Anti-Apoptosis and Autophagy Effects of Melatonin Protect Rat Chondrocytes against Oxidative Stress via Regulation of AMPK/Foxo3 Pathways.<br>Chen Z, Zhao C, Liu P, Huang H, Zhang S, Wang X.<br>Cartilage (2021)<br>PMID 34775836; doi:10.1177/19476035211038748	Controlled animal/mixed preclinical study. Screen-level limitation flag: incomplete reporting of randomization, allocation concealment, blinding, attrition, and prospective sample-size justification.
R0660	2021	Osteoarthritis/cartilage<br>Animal	Chondroprotective effect of melatonin and strontium ranelate in animal model of osteoarthritis.<br>Paulino Silva KM, de Sousa FL, Alves ACB, Rocha PA, da Costa HNAF, Ferreira WR, Reis TS, de Oliveira TKB, Cabral Batista SR, Lapa Neto CJC, Oliveira AG, de Lemos Jordão AJJM.<br>Heliyon (2021)<br>PMID 33912721; doi:10.1016/j.heliyon.2021.e06760	Controlled animal/mixed preclinical study. Screen-level limitation flag: incomplete reporting of randomization, allocation concealment, blinding, attrition, and prospective sample-size justification.
R0774	2019	Osteoarthritis/cartilage<br>Animal	Melatonin Prevents Osteoarthritis-Induced Cartilage Degradation via Targeting MicroRNA-140.<br>Zhang Y, Lin J, Zhou X, Chen X, Chen AC, Pi B, Pan G, Pei M, Yang H, Liu T, He F.<br>Oxidative medicine and cellular longevity (2019)<br>PMID 31915516; doi:10.1155/2019/9705929	Controlled animal/mixed preclinical study. Screen-level limitation flag: incomplete reporting of randomization, allocation concealment, blinding, attrition, and prospective sample-size justification.
R0866	2019	Osteoarthritis/cartilage<br>Animal	Melatonin protects vertebral endplate chondrocytes against apoptosis and calcification via the Sirt1-autophagy pathway.<br>Zhang Z, Lin J, Tian N, Wu Y, Zhou Y, Wang C, Wang Q, Jin H, Chen T, Nisar M, Zheng G, Xu T, Gao W, Zhang X, Wang X.<br>Journal of cellular and molecular medicine (2019)<br>PMID 30353656; doi:10.1111/jcmm.13903	Controlled animal/mixed preclinical study. Screen-level limitation flag: incomplete reporting of randomization, allocation concealment, blinding, attrition, and prospective sample-size justification.
R0946	2017	Osteoarthritis/cartilage<br>Animal	Melatonin inhibits Sirt1-dependent NAMPT and NFAT5 signaling in chondrocytes to attenuate osteoarthritis.<br>Guo JY, Li F, Wen YB, Cui HX, Guo ML, Zhang L, Zhang YF, Guo YJ, Guo YX.<br>Oncotarget (2017)<br>PMID 28915567; doi:10.18632/oncotarget.18356	Controlled animal/mixed preclinical study. Screen-level limitation flag: incomplete reporting of randomization, allocation concealment, blinding, attrition, and prospective sample-size justification.

ID	Year	Domain / stream	Report and identifier	Design and screen-level methodological limitation assessment
R0952	2017	Osteoarthritis/cartilage<br>Animal	Melatonin protects chondrocytes from impairment induced by glucocorticoids via NAD+-dependent SIRT1.<br>Yang W, Kang X, Qin N, Li F, Jin X, Ma Z, Qian Z, Wu S.<br>Steroids (2017)<br>PMID 28803211; doi:10.1016/j.steroids.2017.08.005	Controlled animal/mixed preclinical study. Screen-level limitation flag: incomplete reporting of randomization, allocation concealment, blinding, attrition, and prospective sample-size justification.
R1167	2013	Osteoarthritis/cartilage<br>Animal	Effect of melatonin on the proliferation and differentiation of chondrocytes from rat vertebral body growth plate in vitro.<br>Zhong ZM, Li T, Xu ZX, Meng TT, Zeng JH, Zheng S, Ye WB, Wu Q, Chen JT.<br>International journal of medical sciences (2013)<br>PMID 23983601; doi:10.7150/ijms.5645	Controlled animal/mixed preclinical study. Screen-level limitation flag: incomplete reporting of randomization, allocation concealment, blinding, attrition, and prospective sample-size justification.
R1221	2012	Osteoarthritis/cartilage<br>Animal	Cytoprotective and anti-inflammatory effects of melatonin in hydrogen peroxide-stimulated CHON-001 human chondrocyte cell line and rabbit model of osteoarthritis via the SIRT1 pathway.<br>Lim HD, Kim YS, Ko SH, Yoon IJ, Cho SG, Chun YH, Choi BJ, Kim EC.<br>Journal of pineal research (2012)<br>PMID 22507555; doi:10.1111/j.1600-079x.2012.00991.x	Controlled animal/mixed preclinical study. Screen-level limitation flag: incomplete reporting of randomization, allocation concealment, blinding, attrition, and prospective sample-size justification.
R1271	2010	Osteoarthritis/cartilage<br>Animal	[Effects of melatonin on expression of bone morphogenetic protein 2 and interleukin 1beta in articular cartilage of rat with osteoarthritis].<br>Huang C, Liu J, Xia C, Hu Y.<br>Zhongguo xiu fu chong jian wai ke za zhi = Zhongguo xiufu chongjian waike zazhi = Chinese journal of reparative and reconstructive surgery (2010)<br>PMID 20939479	Controlled animal/mixed preclinical study. Screen-level limitation flag: incomplete reporting of randomization, allocation concealment, blinding, attrition, and prospective sample-size justification.
R0345	2024	Osteoarthritis/cartilage<br>Human	Melatonin is a potential novel analgesic agent for osteoarthritis: Evidence from cohort studies in humans and preclinical research in rats.<br>Li H, Zhou B, Wu J, Zhang Y, Zhang W, Doherty M, Deng X, Wang N, Xie D, Wang Y, Xie H, Li C, Wei J, Lei G, Zeng C.<br>Journal of pineal research (2024)<br>PMID 38348943; doi:10.1111/jpi.12945	Human cohort. Screen-level limitation flag: nonrandomized exposure with potential confounding; outcomes may be indirect.
R0029	2026	Osteoarthritis/cartilage<br>In vitro	3D printed hydrogel scaffold incorporating keratin and melatonin-loaded nanomicelles for cartilage tissue engineering.<br>Hameed AF, Oraibi NS, Mohammed Awad W, Mousa Alawsi S, Al-Musawi MH, Muhammed Muzher H, Hayder Asker M, Sharifianjazi F, Tavamaishvili K, Vesal M, Azad N, Mirhaj M, Tavakoli M.<br>Scientific reports (2026)<br>PMID 42143140; doi:10.1038/s41598-026-53640-y	Controlled in-vitro/cell/tissue study. Screen-level limitation flag: incomplete reporting of concentration justification, independent replication, blinding, and external validity.
R0637	2021	Osteoarthritis/cartilage<br>In vitro	Melatonin contributes to the hypertrophic differentiation of mesenchymal stem cell-derived chondrocytes via activation of the Wnt/β-catenin signaling pathway : Melatonin promotes MSC-derived chondrocytes hypertrophy.<br>Wang X, He T, He L, Yang B, Liu Z, Pang M, Xie P, Zhang L, Rong L, Rong L.<br>Stem cell research & therapy (2021)<br>PMID 34419165; doi:10.1186/s13287-021-02536-x	Controlled in-vitro/cell/tissue study. Screen-level limitation flag: incomplete reporting of concentration justification, independent replication, blinding, and external validity.

ID	Year	Domain / stream	Report and identifier	Design and screen-level methodological limitation assessment
R0739	2020	Osteoarthritis/cartilage<br>In vitro	Injectable gellan gum/lignocellulose nanofibrils hydrogels enriched with melatonin loaded forsterite nanoparticles for cartilage tissue engineering: Fabrication, characterization and cell culture studies.<br>Kouhi M, Varshosaz J, Hashemibeni B, Sarmadi A.<br>Materials science & engineering. C, Materials for biological applications (2020)<br>PMID 32600714; doi:10.1016/j.msec.2020.111114	Controlled in-vitro/cell/tissue study. Screen-level limitation flag: incomplete reporting of concentration justification, independent replication, blinding, and external validity.
R1328	2009	Osteoarthritis/cartilage<br>In vitro	Melatonin enhances cartilage matrix synthesis by porcine articular chondrocytes.<br>Pei M, He F, Wei L, Rawson A.<br>Journal of pineal research (2009)<br>PMID 19054299; doi:10.1111/j.1600-079x.2008.00646.x	Controlled in-vitro/cell/tissue study. Screen-level limitation flag: incomplete reporting of concentration justification, independent replication, blinding, and external validity.
R0180	2025	Perioperative orthopedic care<br>Human	Does Melatonin Improve Sleep Following Primary Total Hip Arthroplasty? A Randomized, Double-Blind, Placebo-Controlled Trial.<br>Haider MA, Lawrence KW, Christensen T, Schwarzkopf R, Macaulay W, Rozell JC.<br>The Journal of arthroplasty (2025)<br>PMID 40383169; doi:10.1016/j.arth.2025.05.038	Human randomized trial. Screen-level limitation flag: small/short trial; incomplete reporting of allocation concealment, multiplicity, or attrition. Outcome is supportive-care or surrogate rather than proof of disease modification.
R0285	2025	Perioperative orthopedic care<br>Human	Evaluation of postoperative delirium in geriatric patients given acetaminophen with and without melatonin: A retrospective cohort study.<br>Muehrcke MN, Blackwelder RS, Weeda ER, Furlough LA, Shelton JE, Brace SM, Bragg SW.<br>International journal of psychiatry in medicine (2025)<br>PMID 39161086; doi:10.1177/00912174241276596	Human cohort. Screen-level limitation flag: nonrandomized exposure with potential confounding; outcomes may be indirect. Outcome is supportive-care or surrogate rather than proof of disease modification.
R0340	2024	Perioperative orthopedic care<br>Human	Does Melatonin Improve Sleep Following Primary Total Knee Arthroplasty? A Randomized, Double-Blind, Placebo-Controlled Trial.<br>Haider MA, Lawrence KW, Christensen T, Schwarzkopf R, Macaulay W, Rozell JC.<br>The Journal of arthroplasty (2024)<br>PMID 38401621; doi:10.1016/j.arth.2024.02.031	Human randomized trial. Screen-level limitation flag: small/short trial; incomplete reporting of allocation concealment, multiplicity, or attrition. Outcome is supportive-care or surrogate rather than proof of disease modification.
R0352	2024	Perioperative orthopedic care<br>Human	The John N. Insall Award: Does Melatonin Improve Subjective Sleep Quality After Total Knee Arthroplasty? A Randomized, Placebo-Controlled Trial.<br>LeBrun DG, Grubel J, Ong J, Chiu YF, Blevins JL, Haas SB, Rodriguez JA, Gausden EB, Cushner FD, Lee GC, Gonzalez Della Valle A, Chalmers BP.<br>The Journal of arthroplasty (2024)<br>PMID 38237875; doi:10.1016/j.arth.2024.01.018	Human randomized trial. Screen-level limitation flag: small/short trial; incomplete reporting of allocation concealment, multiplicity, or attrition. Outcome is supportive-care or surrogate rather than proof of disease modification.
R0394	2023	Perioperative orthopedic care<br>Human	Perioperative Anxiolysis and Analgesic Effect after Premedication with Melatonin and Pregabalin in Total Hip Arthroplasty under Spinal Anaesthesia: A Prospective Comparative Trial.<br>Mishra A, Srivastava VK, Prakash R, Mishra NK, Agarwal J, Kabi S.<br>Advanced biomedical research (2023)<br>PMID 37694243; doi:10.4103/abr.abr_323_22	Human randomized trial. Screen-level limitation flag: small/short trial; incomplete reporting of allocation concealment, multiplicity, or attrition.

ID	Year	Domain / stream	Report and identifier	Design and screen-level methodological limitation assessment
R0485	2022	Perioperative orthopedic care<br>Human	Effectiveness of melatonin treatment for sleep disturbance in orthopaedic trauma patients: A prospective, randomized control trial.<br>Tanner N, Schultz B, Calderon C, Fithian A, Segovia N, Bishop J, Gardner M.<br>Injury (2022)<br>PMID 36424687; doi:10.1016/j.injury.2022.10.011	Human randomized trial. Screen-level limitation flag: small/short trial; incomplete reporting of allocation concealment, multiplicity, or attrition. Outcome is supportive-care or surrogate rather than proof of disease modification.
R0509	2022	Perioperative orthopedic care<br>Human	The effect of melatonin on prevention of postoperative delirium after lower limb fracture surgery in elderly patients: a randomized double blind clinical trial.<br>Fazel MR, Mofidian S, Mahdian M, Akbari H, Razavizadeh MR.<br>International journal of burns and trauma (2022)<br>PMID 36160675	Human randomized trial. Screen-level limitation flag: small/short trial; incomplete reporting of allocation concealment, multiplicity, or attrition. Outcome is supportive-care or surrogate rather than proof of disease modification.
R0532	2022	Perioperative orthopedic care<br>Human	Performance of Melatonin as Prophylaxis in Geriatric Patients with Multifactorial Risk for Postoperative Delirium Development: A Randomized Comparative Study.<br>Mohamed SA, Rady A, Youssry M, Abdelaziz Mohamed MR, Gamal M.<br>Turkish journal of anaesthesiology and reanimation (2022)<br>PMID 35801323; doi:10.5152/tjar.2022.20017	Human randomized trial. Screen-level limitation flag: small/short trial; incomplete reporting of allocation concealment, multiplicity, or attrition. Outcome is supportive-care or surrogate rather than proof of disease modification.
R0572	2022	Perioperative orthopedic care<br>Human	Effect of Preoperative Administration of Oral Melatonin on Pneumatic Tourniquet-Induced Ischemia-Reperfusion Injury in Orthopedic Surgery of Lower Extremities: A Randomized Clinical Trial.<br>Jouybar R, Khademi S, Razmjooie S, Bagheri N.<br>Iranian journal of medical sciences (2022)<br>PMID 35291433; doi:10.30476/ijms.2021.86960.1701	Human randomized trial. Screen-level limitation flag: small/short trial; incomplete reporting of allocation concealment, multiplicity, or attrition.
R0615	2022	Perioperative orthopedic care<br>Human	Melatonin Does Not Improve Sleep Quality in a Randomized Placebo-controlled Trial After Primary Total Joint Arthroplasty.<br>Clarkson SJ, Yayac MF, Rondon AJ, Smith BM, Purtill JJ.<br>The Journal of the American Academy of Orthopaedic Surgeons (2022)<br>PMID 34724457; doi:10.5435/jaaos-d-21-00243	Human randomized trial. Screen-level limitation flag: small/short trial; incomplete reporting of allocation concealment, multiplicity, or attrition. Outcome is supportive-care or surrogate rather than proof of disease modification.
R0974	2017	Perioperative orthopedic care<br>Human	The effect of melatonin on early postoperative cognitive decline in elderly patients undergoing hip arthroplasty: A randomized controlled trial.<br>Fan Y, Yuan L, Ji M, Yang J, Gao D.<br>Journal of clinical anesthesia (2017)<br>PMID 28494914; doi:10.1016/j.jclinane.2017.03.023	Human cohort. Screen-level limitation flag: nonrandomized exposure with potential confounding; outcomes may be indirect.
R1078	2015	Perioperative orthopedic care<br>Human	Impact of Melatonin on Sleep and Pain After Total Knee Arthroplasty Under Regional Anesthesia With Sedation: A Double-Blind, Randomized, Placebo-Controlled Pilot Study.<br>Kirksey MA, Yoo D, Danninger T, Stundner O, Ma Y, Memtsoudis SG.<br>The Journal of arthroplasty (2015)<br>PMID 26173613; doi:10.1016/j.arth.2015.06.034	Human randomized trial. Screen-level limitation flag: small/short trial; incomplete reporting of allocation concealment, multiplicity, or attrition. Outcome is supportive-care or surrogate rather than proof of disease modification.

ID	Year	Domain / stream	Report and identifier	Design and screen-level methodological limitation assessment
R1263	2010	Perioperative orthopedic care<br>Human	Assessment of role of perioperative melatonin in prevention and treatment of postoperative delirium after hip arthroplasty under spinal anesthesia in the elderly.<br>Sultan SS.<br>Saudi journal of anaesthesia (2010)<br>PMID 21189854; doi:10.4103/1658-354x.71132	Human randomized trial. Screen-level limitation flag: small/short trial; incomplete reporting of allocation concealment, multiplicity, or attrition. Outcome is supportive-care or surrogate rather than proof of disease modification.
R0114	2025	Sarcopenia/muscle injury<br>Animal	Melatonin Activates AMPK Pathway to Regulate the Regeneration of Slow Muscle Fibers in Skeletal Muscle Injury Repair.<br>Xie W, Hong Z, Xie Y, Hou K, Li S, Fang M, Guan Y, Zhang Z, Zhang M.<br>FASEB journal : official publication of the Federation of American Societies for Experimental Biology (2025)<br>PMID 41077888; doi:10.1096/fj.202501549rr	Controlled animal/mixed preclinical study. Screen-level limitation flag: incomplete reporting of randomization, allocation concealment, blinding, attrition, and prospective sample-size justification.
R0165	2025	Sarcopenia/muscle injury<br>Animal	Melatonin Mitigates Sarcopenic Obesity via Microbiota and Short-Chain Fatty Acids: Evidence From Epidemiologic and In Vivo Studies.<br>Mo X, Shen L, Wang X, Ni W, Li L, Xia L, Liu H, Cheng R, Wen L, Xu J, Liu L.<br>Journal of cachexia, sarcopenia and muscle (2025)<br>PMID 40511533; doi:10.1002/jcsm.13869	Controlled animal/mixed preclinical study. Screen-level limitation flag: incomplete reporting of randomization, allocation concealment, blinding, attrition, and prospective sample-size justification.
R0216	2025	Sarcopenia/muscle injury<br>Animal	Melatonin Ameliorates Age-Related Sarcopenia via the Gut-Muscle Axis Mediated by Serum Lipopolysaccharide and Metabolites.<br>Zhou LS, Yang Y, Mou L, Xia X, Liu M, Xu LJ, Liu R, Liu JP, Zhang HY, Ao XJ, Liu CJ, Xiao Q, Liu SX.<br>Journal of cachexia, sarcopenia and muscle (2025)<br>PMID 39901379; doi:10.1002/jcsm.13722	Controlled animal/mixed preclinical study. Screen-level limitation flag: incomplete reporting of randomization, allocation concealment, blinding, attrition, and prospective sample-size justification.
R0247	2025	Sarcopenia/muscle injury<br>Animal	Melatonin attenuates sepsis-induced muscle atrophy by regulating the PI3K/Akt signaling pathway.<br>Yao H, Xie Q, Yang Y, Zhou C, Zeng Z, Zhang W.<br>International immunopharmacology (2025)<br>PMID 39602954; doi:10.1016/j.intimp.2024.113619	Controlled animal/mixed preclinical study. Screen-level limitation flag: incomplete reporting of randomization, allocation concealment, blinding, attrition, and prospective sample-size justification.
R0267	2025	Sarcopenia/muscle injury<br>Animal	Melatonin protects against sarcopenia in middle-aged mice.<br>Fang F, Yu P, Sun X, Shen Z, Zhang F, Sun J.<br>Histology and histopathology (2025)<br>PMID 39385610; doi:10.14670/hh-18-814	Controlled animal/mixed preclinical study. Screen-level limitation flag: incomplete reporting of randomization, allocation concealment, blinding, attrition, and prospective sample-size justification.
R0245	2024	Sarcopenia/muscle injury<br>Animal	Melatonin ameliorates age-related sarcopenia by inhibiting fibrogenic conversion of satellite cell.<br>Zhu GZ, Zhao K, Li HZ, Wu DZ, Chen YB, Han D, Gao JW, Chen XY, Yu YP, Huang ZW, Tu C, Zhong ZM.<br>Molecular medicine (Cambridge, Mass.) (2024)<br>PMID 39614149; doi:10.1186/s10020-024-00998-2	Controlled animal/mixed preclinical study. Screen-level limitation flag: incomplete reporting of randomization, allocation concealment, blinding, attrition, and prospective sample-size justification.

ID	Year	Domain / stream	Report and identifier	Design and screen-level methodological limitation assessment
R0366	2024	Sarcopenia/muscle injury<br>Animal	Enhancement of mitochondrial energy metabolism by melatonin promotes vascularized skeletal muscle regeneration in a volumetric muscle loss model.<br>Ge X, Wang C, Yang G, Maimaiti D, Hou M, Liu H, Yang H, Chen X, Xu Y, He F.<br>Free radical biology & medicine (2024)<br>PMID 38008130; doi:10.1016/j.freeradbiomed.2023.11.021	Controlled animal/mixed preclinical study. Screen-level limitation flag: incomplete reporting of randomization, allocation concealment, blinding, attrition, and prospective sample-size justification.
R0403	2023	Sarcopenia/muscle injury<br>Animal	Melatonin Improves Skeletal Muscle Structure and Oxidative Phenotype by Regulating Mitochondrial Dynamics and Autophagy in Zücker Diabetic Fatty Rat.<br>Salagre D, Raya Álvarez E, Cendan CM, Aouichat S, Agil A.<br>Antioxidants (Basel, Switzerland) (2023)<br>PMID 37627494; doi:10.3390/antiox12081499	Controlled animal/mixed preclinical study. Screen-level limitation flag: incomplete reporting of randomization, allocation concealment, blinding, attrition, and prospective sample-size justification.
R0451	2023	Sarcopenia/muscle injury<br>Animal	Melatonin improves muscle injury and differentiation by increasing Pax7 expression.<br>Su CM, Tsai CH, Chen HT, Wu YS, Chang JW, Yang SF, Tang CH.<br>International journal of biological sciences (2023)<br>PMID 36923937; doi:10.7150/ijbs.79169	Controlled animal/mixed preclinical study. Screen-level limitation flag: incomplete reporting of randomization, allocation concealment, blinding, attrition, and prospective sample-size justification.
R0795	2019	Sarcopenia/muscle injury<br>Animal	Myomaker, and Myomixer-Myomerger-Minion modulate the efficiency of skeletal muscle development with melatonin supplementation through Wnt/β-catenin pathway.<br>Chen B, You W, Shan T.<br>Experimental cell research (2019)<br>PMID 31682812; doi:10.1016/j.yexcr.2019.111705	Controlled animal/mixed preclinical study. Screen-level limitation flag: incomplete reporting of randomization, allocation concealment, blinding, attrition, and prospective sample-size justification.
R0843	2019	Sarcopenia/muscle injury<br>Animal	Lack of NLRP3 Inflammasome Activation Reduces Age-Dependent Sarcopenia and Mitochondrial Dysfunction, Favoring the Prophylactic Effect of Melatonin.<br>Sayed RKA, Fernández-Ortiz M, Diaz-Casado ME, Aranda-Martínez P, Fernández-Martínez J, Guerra-Librero A, Escames G, López LC, Alsaadawy RM, Acuña-Castroviejo D.<br>The journals of gerontology. Series A, Biological sciences and medical sciences (2019)<br>PMID 30869745; doi:10.1093/gerona/glz079	Controlled animal/mixed preclinical study. Screen-level limitation flag: incomplete reporting of randomization, allocation concealment, blinding, attrition, and prospective sample-size justification.
R0863	2018	Sarcopenia/muscle injury<br>Animal	Effects of melatonin on oxidative stress parameters and pathohistological changes in rat skeletal muscle tissue following carbon tetrachloride application.<br>Sokolović DT, Lilić L, Milenković V, Stefanović R, Ilić TP, Mekić B, Ilić I, Stojanović NM, Ilić IR.<br>Saudi pharmaceutical journal : SPJ : the official publication of the Saudi Pharmaceutical Society (2018)<br>PMID 30416361; doi:10.1016/j.jsps.2018.05.013	Controlled animal/mixed preclinical study. Screen-level limitation flag: incomplete reporting of randomization, allocation concealment, blinding, attrition, and prospective sample-size justification.
R0909	2018	Sarcopenia/muscle injury<br>Animal	The Protective Effect of Melatonin Against Age-Associated, Sarcopenia-Dependent Tubular Aggregate Formation, Lactate Depletion, and Mitochondrial Changes.<br>Sayed RKA, Fernández-Ortiz M, Diaz-Casado ME, Rusanova I, Rahim I, Escames G, López LC, Mokhtar DM, Acuña-Castroviejo D.<br>The journals of gerontology. Series A, Biological sciences and medical sciences (2018)<br>PMID 29562315; doi:10.1093/gerona/gly059	Controlled animal/mixed preclinical study. Screen-level limitation flag: incomplete reporting of randomization, allocation concealment, blinding, attrition, and prospective sample-size justification.

ID	Year	Domain / stream	Report and identifier	Design and screen-level methodological limitation assessment
R1131	2014	Sarcopenia/muscle injury<br>Animal	Melatonin prevents mitochondrial dysfunction and insulin resistance in rat skeletal muscle.<br>Teodoro BG, Baraldi FG, Sampaio IH, Bomfim LH, Queiroz AL, Passos MA, Carneiro EM, Alberici LC, Gomis R, Amaral FG, Cipolla-Neto J, Araújo MB, Lima T, Akira Uyemura S, Silveira LR, Vieira E.<br>Journal of pineal research (2014)<br>PMID 24981026; doi:10.1111/jpi.12157	Controlled animal/mixed preclinical study. Screen-level limitation flag: incomplete reporting of randomization, allocation concealment, blinding, attrition, and prospective sample-size justification.
R1283	2011	Sarcopenia/muscle injury<br>Animal	Melatonin attenuates I/R-induced mitochondrial dysfunction in skeletal muscle.<br>Wang WZ, Fang XH, Stephenson LL, Zhang X, Khiabani KT, Zamboni WA.<br>The Journal of surgical research (2011)<br>PMID 20421117; doi:10.1016/j.jss.2010.01.019	Controlled animal/mixed preclinical study. Screen-level limitation flag: incomplete reporting of randomization, allocation concealment, blinding, attrition, and prospective sample-size justification.
R1269	2010	Sarcopenia/muscle injury<br>Animal	[Protective effects of melatonin on ischemia-reperfusion injury of skeletal muscle].<br>Erdem M, Bostan B, Güneş T, Özkan F, Sen C, Özyurt H, Köseoğlu RD, Erdoğan H.<br>Eklem hastaliklari ve cerrahisi = Joint diseases & related surgery (2010)<br>PMID 21067499	Controlled animal/mixed preclinical study. Screen-level limitation flag: incomplete reporting of randomization, allocation concealment, blinding, attrition, and prospective sample-size justification.
R1311	2009	Sarcopenia/muscle injury<br>Animal	Melatonin prevents oxidative stress-mediated mitochondrial permeability transition and death in skeletal muscle cells.<br>Hibaoui Y, Roulet E, Ruegg UT.<br>Journal of pineal research (2009)<br>PMID 19664004; doi:10.1111/j.1600-079x.2009.00707.x	Controlled animal/mixed preclinical study. Screen-level limitation flag: incomplete reporting of randomization, allocation concealment, blinding, attrition, and prospective sample-size justification.
R1392	2006	Sarcopenia/muscle injury<br>Animal	Melatonin inhibits the expression of the inducible isoform of nitric oxide synthase and nuclear factor kappa B activation in rat skeletal muscle.<br>Alonso M, Collado PS, González-Gallego J.<br>Journal of pineal research (2006)<br>PMID 16842535; doi:10.1111/j.1600-079x.2006.00323.x	Controlled animal/mixed preclinical study. Screen-level limitation flag: incomplete reporting of randomization, allocation concealment, blinding, attrition, and prospective sample-size justification.
R1408	2006	Sarcopenia/muscle injury<br>Animal	Melatonin counteracts inducible mitochondrial nitric oxide synthase-dependent mitochondrial dysfunction in skeletal muscle of septic mice.<br>Escames G, López LC, Tapias V, Utrilla P, Reiter RJ, Hitos AB, León J, Rodríguez MI, Acuña-Castroviejo D.<br>Journal of pineal research (2006)<br>PMID 16313501; doi:10.1111/j.1600-079x.2005.00281.x	Controlled animal/mixed preclinical study. Screen-level limitation flag: incomplete reporting of randomization, allocation concealment, blinding, attrition, and prospective sample-size justification.
R1414	2005	Sarcopenia/muscle injury<br>Animal	Melatonin protects against ischemia/reperfusion injury in skeletal muscle.<br>Erkanli K, Kayalar N, Erkanli G, Ercan F, Sener G, Kirali K.<br>Journal of pineal research (2005)<br>PMID 16150103; doi:10.1111/j.1600-079x.2005.00240.x	Controlled animal/mixed preclinical study. Screen-level limitation flag: incomplete reporting of randomization, allocation concealment, blinding, attrition, and prospective sample-size justification.

ID	Year	Domain / stream	Report and identifier	Design and screen-level methodological limitation assessment
R1417	2005	Sarcopenia/muscle injury<br>Animal	Microcirculatory effects of melatonin in rat skeletal muscle after prolonged ischemia.<br>Wang WZ, Fang XH, Stephenson LL, Baynosa RC, Khiabani KT, Zamboni WA.<br>Journal of pineal research (2005)<br>PMID 15978058; doi:10.1111/j.1600-079x.2005.00215.x	Controlled animal/mixed preclinical study. Screen-level limitation flag: incomplete reporting of randomization, allocation concealment, blinding, attrition, and prospective sample-size justification.
R1429	2004	Sarcopenia/muscle injury<br>Animal	The effect of melatonin on plasma oxidant-antioxidant skeletal muscle reperfusion injury in rats.<br>Halici M, Narin F, Turk CY, Saraymen R, Baykan A, Kabak S.<br>The Journal of international medical research (2004)<br>PMID 15458282; doi:10.1177/147323000403200507	Controlled animal/mixed preclinical study. Screen-level limitation flag: incomplete reporting of randomization, allocation concealment, blinding, attrition, and prospective sample-size justification.
R1443	2003	Sarcopenia/muscle injury<br>Animal	Effects of melatonin on skeletal muscle of rats with experimental hyperthyroidism.<br>Oner J, Ozan E.<br>Endocrine research (2003)<br>PMID 14682473; doi:10.1081/erc-120026950	Controlled animal/mixed preclinical study. Screen-level limitation flag: incomplete reporting of randomization, allocation concealment, blinding, attrition, and prospective sample-size justification.
R0834	2018	Sarcopenia/muscle injury<br>Human	Is a Combination of Melatonin and Amino Acids Useful to Sarcopenic Elderly Patients? A Randomized Trial.<br>Rondanelli M, Peroni G, Gasparri C, Infantino V, Nichetti M, Cuzzoni G, Spadaccini D, Perna S.<br>Geriatrics (Basel, Switzerland) (2018)<br>PMID 31023972; doi:10.3390/geriatrics4010004	Human randomized trial. Screen-level limitation flag: small/short trial; incomplete reporting of allocation concealment, multiplicity, or attrition.
R0954	2017	Sarcopenia/muscle injury<br>In vitro	Melatonin prevents mitochondrial dysfunctions and death in differentiated skeletal muscle cells.<br>Salucci S, Battistelli M, Baldassarri V, Burini D, Falcieri E, Burattini S.<br>Microscopy research and technique (2017)<br>PMID 28742227; doi:10.1002/jemt.22914	Controlled in-vitro/cell/tissue study. Screen-level limitation flag: incomplete reporting of concentration justification, independent replication, blinding, and external validity.
R0086	2025	Spinal cord injury<br>Animal	Ameliorative effects of dexpanthenol and/or melatonin application in experimental spinal cord injury.<br>Korkmaz MF, Cigremis Y, Eroglu A, Yalcinkaya B, Ozer OF, Cobanoglu B, Baysal B, Ankarali H, Taslak H, Bozduman O.<br>The journal of spinal cord medicine (2025)<br>PMID 41358999; doi:10.1080/10790268.2025.2580127	Controlled animal/mixed preclinical study. Screen-level limitation flag: incomplete reporting of randomization, allocation concealment, blinding, attrition, and prospective sample-size justification.
R0099	2025	Spinal cord injury<br>Animal	Effect of melatonin loaded chitosan hydrogel on rat spinal cord injury.<br>Afroozi F, Asghari A, Abedi G, Mortazavi P, Hoseinzadeh HU.<br>Veterinary research forum : an international quarterly journal (2025)<br>PMID 41195216; doi:10.30466/vrf.2025.2043413.4497	Controlled animal/mixed preclinical study. Screen-level limitation flag: incomplete reporting of randomization, allocation concealment, blinding, attrition, and prospective sample-size justification.

ID	Year	Domain / stream	Report and identifier	Design and screen-level methodological limitation assessment
R0360	2024	Spinal cord injury<br>Animal	Melatonin exerts neuroprotective effects in mice with spinal cord injury by activating the Nrf2/Keap1 signaling pathway via the MT2 receptor.<br>Yan L, Han X, Zhang M, Kou H, Liu H, Cheng T.<br>Experimental and therapeutic medicine (2024)<br>PMID 38125360; doi:10.3892/etm.2023.12325	Controlled animal/mixed preclinical study. Screen-level limitation flag: incomplete reporting of randomization, allocation concealment, blinding, attrition, and prospective sample-size justification.
R0478	2023	Spinal cord injury<br>Animal	Melatonin promotes microglia toward anti-inflammatory phenotype after spinal cord injury.<br>Guo Y, Zhang P, Zhao H, Xu C, Lin S, Mei X, Tian H.<br>International immunopharmacology (2023)<br>PMID 36538849; doi:10.1016/j.intimp.2022.109599	Controlled animal/mixed preclinical study. Screen-level limitation flag: incomplete reporting of randomization, allocation concealment, blinding, attrition, and prospective sample-size justification.
R0487	2022	Spinal cord injury<br>Animal	Effects of melatonin-pretreated adipose-derived mesenchymal stem cells (MSC) in an animal model of spinal cord injury.<br>Naeimi A, Zaminy A, Amini N, Balabandi R, Golipoor Z.<br>BMC neuroscience (2022)<br>PMID 36384473; doi:10.1186/s12868-022-00752-6	Controlled animal/mixed preclinical study. Screen-level limitation flag: incomplete reporting of randomization, allocation concealment, blinding, attrition, and prospective sample-size justification.
R0510	2022	Spinal cord injury<br>Animal	Melatonin Attenuates Spinal Cord Injury in Mice by Activating the Nrf2/ARE Signaling Pathway to Inhibit the NLRP3 Inflammasome.<br>Wang H, Wang H, Huang H, Qu Z, Ma D, Dang X, Dong Q.<br>Cells (2022)<br>PMID 36139384; doi:10.3390/cells11182809	Controlled animal/mixed preclinical study. Screen-level limitation flag: incomplete reporting of randomization, allocation concealment, blinding, attrition, and prospective sample-size justification.
R0697	2021	Spinal cord injury<br>Animal	Intrathecal Administration of Melatonin Ameliorates the Neuroinflammation- Mediated Sensory and Motor Dysfunction in A Rat Model of Compression Spinal Cord Injury.<br>Fakhri S, Kiani A, Jalili C, Abbaszadeh F, Piri S, Farzaei MH, Rastegari-Pouyani M, Mohammadi-Noori E, Khan H.<br>Current molecular pharmacology (2021)<br>PMID 33380311; doi:10.2174/1874467213666201230101811	Controlled animal/mixed preclinical study. Screen-level limitation flag: incomplete reporting of randomization, allocation concealment, blinding, attrition, and prospective sample-size justification.
R0775	2020	Spinal cord injury<br>Animal	Melatonin exerts neuroprotective effects by attenuating astro- and microgliosis and suppressing inflammatory response following spinal cord injury.<br>Yang Z, Bao Y, Chen W, He Y.<br>Neuropeptides (2020)<br>PMID 31902595; doi:10.1016/j.npep.2019.102002	Controlled animal/mixed preclinical study. Screen-level limitation flag: incomplete reporting of randomization, allocation concealment, blinding, attrition, and prospective sample-size justification.
R0821	2019	Spinal cord injury<br>Animal	Melatonin Enhances Autophagy and Reduces Apoptosis to Promote Locomotor Recovery in Spinal Cord Injury via the PI3K/AKT/mTOR Signaling Pathway.<br>Li Y, Guo Y, Fan Y, Tian H, Li K, Mei X.<br>Neurochemical research (2019)<br>PMID 31325156; doi:10.1007/s11064-019-02838-w	Controlled animal/mixed preclinical study. Screen-level limitation flag: incomplete reporting of randomization, allocation concealment, blinding, attrition, and prospective sample-size justification.

ID	Year	Domain / stream	Report and identifier	Design and screen-level methodological limitation assessment
R0836	2019	Spinal cord injury<br>Animal	Melatonin improves functional recovery in female rats after acute spinal cord injury by modulating polarization of spinal microglial/macrophages.<br>Zhang Y, Liu Z, Zhang W, Wu Q, Zhang Y, Liu Y, Guan Y, Chen X.<br>Journal of neuroscience research (2019)<br>PMID 31006904; doi:10.1002/jnr.24409	Controlled animal/mixed preclinical study. Screen-level limitation flag: incomplete reporting of randomization, allocation concealment, blinding, attrition, and prospective sample-size justification.
R0871	2019	Spinal cord injury<br>Animal	Melatonin ameliorates spinal cord injury by suppressing the activation of inflammasomes in rats.<br>Xu G, Shi D, Zhi Z, Ao R, Yu B.<br>Journal of cellular biochemistry (2019)<br>PMID 30257055; doi:10.1002/jcb.27794	Controlled animal/mixed preclinical study. Screen-level limitation flag: incomplete reporting of randomization, allocation concealment, blinding, attrition, and prospective sample-size justification.
R0978	2017	Spinal cord injury<br>Animal	Melatonin Inhibits Neural Cell Apoptosis and Promotes Locomotor Recovery via Activation of the Wnt/β-Catenin Signaling Pathway After Spinal Cord Injury.<br>Shen Z, Zhou Z, Gao S, Guo Y, Gao K, Wang H, Dang X.<br>Neurochemical research (2017)<br>PMID 28417262; doi:10.1007/s11064-017-2251-7	Controlled animal/mixed preclinical study. Screen-level limitation flag: incomplete reporting of randomization, allocation concealment, blinding, attrition, and prospective sample-size justification.
R1008	2017	Spinal cord injury<br>Animal	Melatonin prevents blood vessel loss and neurological impairment induced by spinal cord injury in rats.<br>Jing Y, Bai F, Chen H, Dong H.<br>The journal of spinal cord medicine (2017)<br>PMID 27735218; doi:10.1080/10790268.2016.1227912	Controlled animal/mixed preclinical study. Screen-level limitation flag: incomplete reporting of randomization, allocation concealment, blinding, attrition, and prospective sample-size justification.
R1009	2017	Spinal cord injury<br>Animal	PPAR-α Modulates the Anti-Inflammatory Effect of Melatonin in the Secondary Events of Spinal Cord Injury.<br>Paterniti I, Campolo M, Cordaro M, Impellizzeri D, Siracusa R, Crupi R, Esposito E, Cuzzocrea S.<br>Molecular neurobiology (2017)<br>PMID 27686077; doi:10.1007/s12035-016-0131-9	Controlled animal/mixed preclinical study. Screen-level limitation flag: incomplete reporting of randomization, allocation concealment, blinding, attrition, and prospective sample-size justification.
R1007	2016	Spinal cord injury<br>Animal	Effects of melatonin on severe crush spinal cord injury-induced reactive astrocyte and scar formation.<br>Krityakiarana W, Sompup K, Jongkamonwiwat N, Mukda S, Pinilla FG, Govitrapong P, Phansuwan-Pujito P.<br>Journal of neuroscience research (2016)<br>PMID 27717042; doi:10.1002/jnr.23930	Controlled animal/mixed preclinical study. Screen-level limitation flag: incomplete reporting of randomization, allocation concealment, blinding, attrition, and prospective sample-size justification.
R1086	2015	Spinal cord injury<br>Animal	Anti-edema effect of melatonin on spinal cord injury in rats.<br>Liu X, Wang Y, Yang J, Liu Y, Zhou D, Hou M, Xiang L.<br>Biomedical papers of the Medical Faculty of the University Palacky, Olomouc, Czechoslovakia (2015)<br>PMID 25916278; doi:10.5507/bp.2015.012	Controlled animal/mixed preclinical study. Screen-level limitation flag: incomplete reporting of randomization, allocation concealment, blinding, attrition, and prospective sample-size justification.

ID	Year	Domain / stream	Report and identifier	Design and screen-level methodological limitation assessment
R1112	2014	Spinal cord injury<br>Animal	Melatonin treatment protects against acute spinal cord injury-induced disruption of blood spinal cord barrier in mice.<br>Wu Q, Jing Y, Yuan X, Zhang X, Li B, Liu M, Wang B, Li H, Liu S, Xiu R.<br>Journal of molecular neuroscience : MN (2014)<br>PMID 25303856; doi:10.1007/s12031-014-0430-4	Controlled animal/mixed preclinical study. Screen-level limitation flag: incomplete reporting of randomization, allocation concealment, blinding, attrition, and prospective sample-size justification.
R1138	2014	Spinal cord injury<br>Animal	Melatonin improves functional outcome via inhibition of matrix metalloproteinases-9 after photothrombotic spinal cord injury in rats.<br>Piao MS, Lee JK, Jang JW, Hur H, Lee SS, Xiao L, Kim HS.<br>Acta neurochirurgica (2014)<br>PMID 24879621; doi:10.1007/s00701-014-2119-4	Controlled animal/mixed preclinical study. Screen-level limitation flag: incomplete reporting of randomization, allocation concealment, blinding, attrition, and prospective sample-size justification.
R1290	2010	Spinal cord injury<br>Animal	Synergistic effect of melatonin on exercise-induced neuronal reconstruction and functional recovery in a spinal cord injury animal model.<br>Park K, Lee Y, Park S, Lee S, Hong Y, Kil Lee S, Hong Y.<br>Journal of pineal research (2010)<br>PMID 20210855; doi:10.1111/j.1600-079x.2010.00751.x	Controlled animal/mixed preclinical study. Screen-level limitation flag: incomplete reporting of randomization, allocation concealment, blinding, attrition, and prospective sample-size justification.
R1352	2008	Spinal cord injury<br>Animal	Melatonin regulates matrix metalloproteinases after traumatic experimental spinal cord injury.<br>Esposito E, Genovese T, Caminiti R, Bramanti P, Meli R, Cuzzocrea S.<br>Journal of pineal research (2008)<br>PMID 18298463; doi:10.1111/j.1600-079x.2008.00569.x	Controlled animal/mixed preclinical study. Screen-level limitation flag: incomplete reporting of randomization, allocation concealment, blinding, attrition, and prospective sample-size justification.
R1354	2008	Spinal cord injury<br>Animal	Effects of melatonin on ischemic spinal cord injury caused by aortic cross clamping in rabbits.<br>Korkmaz A, Oyar EO, Kardeş O, Omeroğlu S.<br>Current neurovascular research (2008)<br>PMID 18289021; doi:10.2174/156720208783565681	Controlled animal/mixed preclinical study. Screen-level limitation flag: incomplete reporting of randomization, allocation concealment, blinding, attrition, and prospective sample-size justification.
R1360	2008	Spinal cord injury<br>Animal	Melatonin attenuates calpain upregulation, axonal damage and neuronal death in spinal cord injury in rats.<br>Samantaray S, Sribnick EA, Das A, Knaryan VH, Matzelle DD, Yallapragada AV, Reiter RJ, Ray SK, Banik NL.<br>Journal of pineal research (2008)<br>PMID 18086148; doi:10.1111/j.1600-079x.2007.00534.x	Controlled animal/mixed preclinical study. Screen-level limitation flag: incomplete reporting of randomization, allocation concealment, blinding, attrition, and prospective sample-size justification.
R1410	2005	Spinal cord injury<br>Animal	Dose-dependent neuroprotective effects of melatonin on experimental spinal cord injury in rats.<br>Gül S, Celik SE, Kalayci M, Taşyürekli M, Cokar N, Bilge T.<br>Surgical neurology (2005)<br>PMID 16231427; doi:10.1016/j.surneu.2005.03.036	Controlled animal/mixed preclinical study. Screen-level limitation flag: incomplete reporting of randomization, allocation concealment, blinding, attrition, and prospective sample-size justification.

ID	Year	Domain / stream	Report and identifier	Design and screen-level methodological limitation assessment
R1505	2000	Spinal cord injury<br>Animal	Comparison of the effects of melatonin and methylprednisolone in experimental spinal cord injury.<br>Kaptanoglu E, Tuncel M, Palaoglu S, Konan A, Demirpençe E, Kilinç K.<br>Journal of neurosurgery (2000)<br>PMID 10879762; doi:10.3171/spi.2000.93.1.0077	Controlled animal/mixed preclinical study. Screen-level limitation flag: incomplete reporting of randomization, allocation concealment, blinding, attrition, and prospective sample-size justification.
R1512	2000	Spinal cord injury<br>Animal	Potent protective effects of melatonin on experimental spinal cord injury.<br>Fujimoto T, Nakamura T, Ikeda T, Takagi K.<br>Spine (2000)<br>PMID 10751286; doi:10.1097/00007632-200004010-00003	Controlled animal/mixed preclinical study. Screen-level limitation flag: incomplete reporting of randomization, allocation concealment, blinding, attrition, and prospective sample-size justification.
R0289	2024	Tendon/ligament disorders<br>Animal	Melatonin supplementation counteracts fiber loss in knee ligaments of diabetes-induced rats.<br>Adamska O, Wnuk A, Kamińska A, Poniatowska M, Maciąg B, Kamiński M, Stolarczyk A, Matin M, Atanasov AG, Łapiński M, Jóźwik A.<br>Frontiers in pharmacology (2024)<br>PMID 39135805; doi:10.3389/fphar.2024.1399719	Controlled animal/mixed preclinical study. Screen-level limitation flag: incomplete reporting of randomization, allocation concealment, blinding, attrition, and prospective sample-size justification.
R0679	2021	Tendon/ligament disorders<br>Animal	Melatonin Promotes Heterotopic Ossification Through Regulation of Endothelial-Mesenchymal Transition in Injured Achilles Tendons in Rats.<br>Zhang J, Tang J, Liu J, Yan B, Yan B, Huang M, Zhang Z, Wang L.<br>Frontiers in cell and developmental biology (2021)<br>PMID 33644068; doi:10.3389/fcell.2021.629274	Controlled animal/mixed preclinical study. Screen-level limitation flag: incomplete reporting of randomization, allocation concealment, blinding, attrition, and prospective sample-size justification. Adverse/context-dependent ossification signal.
R0802	2019	Tendon/ligament disorders<br>Animal	Pro-chondrogenic and immunomodulatory melatonin-loaded electrospun membranes for tendon-to-bone healing.<br>Song W, Ma Z, Wang C, Li H, He Y, He Y.<br>Journal of materials chemistry. B (2019)<br>PMID 31588948; doi:10.1039/c9tb01516g	Controlled animal/mixed preclinical study. Screen-level limitation flag: incomplete reporting of randomization, allocation concealment, blinding, attrition, and prospective sample-size justification.

S4. Interpretive Limitations

This appendix documents a transparent post hoc systematic reconstruction undertaken during peer review. It does not imply prospective registration, duplicate independent screening, uniform full-text access, or formal risk-of-bias or certainty assessment. Complete articles were consulted where accessible; otherwise eligibility and data ascertainment were limited to indexed title, abstract, and metadata, which was not treated as equivalent to full-text assessment. Because complete-article access was not prospectively logged, exact accessed and not-accessed counts cannot be reconstructed reliably. The evidence map contains screen-level methodological limitation flags based on accessible information. Selected domains from SciRAP, SYRCLE, RoB 2, and ROBINS-I informed the flags; the tools were not applied in full, and no study-specific overall risk-of-bias judgment or composite score was assigned. Registry records without peer-reviewed results were mapped separately and were not counted as efficacy evidence.

S5. Registration, Protocol, and Amendments

The review was not registered, and no standalone protocol was prepared. The manuscript was originally submitted as a narrative review. During peer review, before the updated search of 6 August 2026 was run, the PICOS criteria, evidence streams, extraction domains, screen-level methodological limitation assessment approach, and qualitative synthesis plan were specified. The table below records the principal amendments transparently rather than implying

prospective registration. Later editorial revisions did not alter the 3,201 identified records, 1,646 screened records, 437 report-level eligibility decisions based on accessible information, or 208 included reports.

**Element**	**Original approach**	**Revised approach**	**Reason**
Review classification	Narrative review	Systematic review with qualitative synthesis	Electronic searching and explicit eligibility required full systematic-review reporting.
Reporting framework	Narrative methods	PRISMA 2020 plus SWiM	To report selection and non-meta-analytic synthesis transparently.
Search sources and date	PubMed, Scopus, and Web of Science; no final date	Verified PubMed/MEDLINE and Europe PMC searches through 6 August 2026; ClinicalTrials.gov mapped separately	Only rerun and documented sources were retained.
Eligibility	Broad orthopedic relevance	Prespecified multistream PICOS and explicit report-status criteria	To reduce subjective selection.
Screen-level methodological limitations	Not reported	Screen-level limitation flags informed by selected design-relevant domains; tools not applied in full	To describe methodological limitations without implying a complete formal risk-of-bias assessment.
Synthesis	Narrative summary	Structured qualitative synthesis; no pooling	Clinical and methodological heterogeneity prevented defensible meta-analysis.
Screening workflow	Not specified	One reviewer plus a complete consistency pass; no automation	Transparent description of the post hoc reconstruction without inventing duplicate review.
